# Calpain and Reactive Oxygen Species Targets Bax for Mitochondrial Permeabilisation and Caspase Activation in Zerumbone Induced Apoptosis

**DOI:** 10.1371/journal.pone.0059350

**Published:** 2013-04-09

**Authors:** Praveen K. Sobhan, Mahendra Seervi, Lokesh Deb, Saneesh Varghese, Anjana Soman, Jeena Joseph, Krupa Ann Mathew, Godi Raghu, George Thomas, Sreekumar E, Manjula S, Santosh Kumar T. R

**Affiliations:** 1 Cancer Research Program, Rajiv Gandhi Centre for Biotechnology, Thiruvananthapuram, Kerala, India; 2 Institute of Bioresource and Sustainable Development, Imphal, Manipur, India; 3 Spice Genomics, Rajiv Gandhi Centre for Biotechnology, Thiruvananthapuram, Kerala, India; 4 Viral Disease Biology Program, Rajiv Gandhi Centre for Biotechnology, Thiruvananthapuram, Kerala, India; Juntendo University School of Medicine, Japan

## Abstract

Fluorescent protein based signaling probes are emerging as valuable tools to study cell signaling because of their ability to provide spatio- temporal information in non invasive live cell mode. Previously, multiple fluorescent protein probes were employed to characterize key events of apoptosis in diverse experimental systems. We have employed a live cell image based approach to visualize the key events of apoptosis signaling induced by zerumbone, the active principle from ginger *Zingiber zerumbet*, in cancer cells that enabled us to analyze prominent apoptotic changes in a hierarchical manner with temporal resolution. Our studies substantiate that mitochondrial permeabilisation and cytochrome c dependent caspase activation dominate in zerumbone induced cell death. Bax activation, the essential and early event of cell death, is independently activated by reactive oxygen species as well as calpains. Zerumbone failed to induce apoptosis or mitochondrial permeabilisation in Bax knockout cells and over-expression of Bax enhanced cell death induced by zerumbone confirming the essential role of Bax for mitochondrial permeabilsation. Simultaneous inhibition of reactive oxygen species and calpain is required for preventing Bax activation and cell death. However, apoptosis induced by zerumbone was prevented in Bcl 2 and Bcl-XL over-expressing cells, whereas more protection was afforded by Bcl 2 specifically targeted to endoplasmic reticulum. Even though zerumbone treatment down-regulated survival proteins such as XIAP, Survivin and Akt, it failed to affect the pro-apoptotic proteins such as PUMA and BIM. Multiple normal diploid cell lines were employed to address cytotoxic activity of zerumbone and, in general, mammary epithelial cells, endothelial progenitor cells and smooth muscle cells were relatively resistant to zerumbone induced cell death with lesser ROS accumulation than cancer cells.

## Introduction

Most cytotoxic and antitumor agents are non-selective and kill normal proliferating cells also. Identification of active cancer-specific compounds remains as a priority area in drug screening and drug discovery efforts. In the recent past, several natural products were identified as potential hits that selectively target cancer cells sparing normal proliferating cells. Such compounds even though less potent and therapeutically not so active, may form basis for further work to identify cancer specific targets. Recently, piperlogumine was shown to have cancer selective activity by modulating the redox status of the cells [1]. Similarly, zerumbone isolated from Zingiber was reported to attack cancer cells specifically [2]. Subsequent studies identified its ability to modulate NF-κB regulated genes and activation of death receptors for its activity [3], [4]. Potential anti-metastatic activity for zerumbone was shown in tumor models that involve CXCR4 suppression by zerumbone [5]. Even though a wide variety of biological properties including antioxidant, anti-inflammatory and antiviral activities were reported for zerumbone, its important target for mediating cell death and the sequence of apoptotic events triggered by zerumbone remain to be defined.

In order to identify the key signaling events that contribute to cancer cell death by zerumbone, we have utilized multiple live cell image based tools to monitor important events of apoptosis such as mitochondrial permeabilisation, Bax activation, cytochrome c release and caspase activation with high temporal resolution. This approach enabled us to systematically monitor the apoptotic events and identify the key triggers that contribute to zerumbone induced cell death. Additionally, the results indicate that zerumbone induced cell death involves calpain and ROS mediated Bax activation and that Bcl 2 localized at endoplasmic reticulum completely prevents cell death induced by zerumbone. Our work also identified the important anti-apoptotic proteins targeted by zerumbone and the enhanced protective role of Bcl 2 targeted to Endoplasmic Reticulum (ER) on these targets. Here, we demonstrate the potential application of genetically encoded fluorescent probes expressing stable cells to define the hierarchical events of programmed cell death from the initiation phase upstream of mitochondria to the execution phase of caspase activation with high temporal resolution.

## Materials and Methods

### Cells, cell lines and maintenance

Human umbilical cord blood endothelial progenitor cells, human mammary epithelial cells and human smooth muscle cells were procured from Lonza and maintained as per the supplier's protocol. Human umbilical cord vein endothelial cells were isolated and characterized as described earlier [6]. Colon cancer cell lines HCT116 and HCT116Bax KO were kindly provided by Dr. Bert Vogelstein (John Hopkins School of Medicine, Baltimore) and maintained in Mc.Coys Medium supplemented with 10% FBS [7]. All other cancer cell lines used were procured either from NCCS, Pune or ATCC, USA. In general, cancer cells were maintained in DMEM (Invitrogen, USA) containing 10% FBS.

### Analysis of reactive oxygen species (ROS)

For detection of intracellular ROS generation, ROS specific fluorescent probe CM-H_2_DCF-DA (Invitrogen) was employed as per the manufacturer's protocol. Briefly, after treating the cells with indicated concentration of drug, the cells were harvested by mild trypsinisation and immediately washed with serum containing medium. Then the cells were treated with CM-H_2_DCF-DA (10 μM) containing serum free OptiMEM medium and analysed by flow cytometry (FACSAria) after 30 minutes of incubation at 37°C. For analyzing intracellular ROS in EGFP transgene expressing cells, Cell ROX Red (Molecular Probes) was used as per the instruction from the manufacturer. Briefly after indicated treatment the cells were trypsinized and stained with 5 µM of Cell ROX Red for 20 minutes at 37°C. Then the cells were analysed using flow cytometer (FACSAria) and signals were collected in APC channel.

### Expression vectors and transfections

The following expression vectors were used for generating stable cell lines. The expression vectors pcDNA3 ECFP-DEVD-EYFP (caspase sensor FRET probe), EGFP-Cyt.c, Bax-EGFP, EGFP-WtBcl2, EGFP-Bcl-XL, EGFP–ERBcl2, pcDNA3 caspase 3 were described previously [8], [9], [10]. The cells were transfected with the expression vector using lipofectamine LTX as per the manufacturer's instruction. Stably expressing clones were generated by selecting the cells in 800 μg/ml of G418 (Invitrogen, Carlsbad, CA) containing medium for 30–40 days. For detecting intracellular calcium and ER calcium, MCF-7 cells were transfected with pcDNA3 YR6 or pcDNA3 D1ER followed by selection for 2 weeks and sorting by flow cytometry for enrichment of cells expressing moderate level of fluorescent proteins, optimal for imaging as described previously [11].

### Determination of conformational activation of Bax and Bak by flow cytometry

The cells after indicated treatment were trypsinized and fixed with 3.7% paraformaldehyde in 1X PBS. The cells were permeabilized with 0.001% CHAPS buffer for 10 min and incubated with a primary antibody that recognizes conformationally active Bak (Calbiochem, TC-100) or Bax (6A7) as described previously [9]. FITC conjugated secondary antibody was used to generate the signal. Total fluorescence intensity from 10,000 cells was read out by FACSAria.

### Analysis of calpain activity by flow cytometry

Calpain activity was measured by using a fluorescent calpain substrate t-BOC-l-leucyl-methionine amide by FACS. Non-fluorescent t-BOC-l-leucyl-methionine amide freely diffuses into the cell and becomes membrane-impermeant after being conjugated to a thiol. Cleavage of t-BOC-thiol by calpain results in the release of fluorescent 7-amino-4-methylcoumarin-thiol (MAC-thiol). The cells were incubated with the fluorescent labeled substrate t-BOC-l-leucyl-methionine amide (10 µM) for 30 minutes at 37°C. The cells were subsequently trypsinized and fluorescence from 10,000 cells was collected at 405/30 nm in 377 nm laser path using FACS Aria II.

### Western blot

The cells treated with or without the drug were washed with 1X PBS and lysed in ice-cold phospho lysis buffer supplemented with protease inhibitors as described [9]. After lysis, the suspension was centrifuged at 12,000rpm for 12 minutes and the supernatant containing the whole cell proteins were immediately used for estimation of protein using Bradford reagent. Proteins were denatured and resolved by SDS-PAGE. The separated proteins were electro blotted on to nitrocellulose membrane using a Bio-Rad Mini PROTEAN western blot apparatus. The blotted membrane was blocked with 5% non-fat milk in Tris buffered saline (TBS) containing 0.2% Tween-20 for 1 h at room temperature. Specific proteins were detected by incubating overnight with appropriate primary antibody in TBST buffer containing 3% BSA at 4°C followed by appropriate secondary antibody conjugated with HRP. The blots were further processed for detecting the signal using ECL reagents (Amersham). The antibodies used and their respective dilutions are given in supplementary methods ( Table S1).

### Live cell ratio imaging by microscopy and pathway Bio-Imager

For live cell imaging, cells were seeded on chambered cover glass (Lab-TekTM, Nunc, NY) and allowed to grow for 24 h. The drug treated cells were maintained on a stage incubation chamber (Okolab, Italy) placed on the stage of microscope (Nikon, TE2000) at 37°C temperature and 5% CO_2_ throughout the imaging period. For microscopic ECFP/EFYP FRET ratio imaging, cells were excited with excitation filter of 438±24 and dual emission was collected with 483±32 and 542±27 using the dichroic 458LP. The excitation and emission filter wheels were independently controlled through NIS element software in automated mode. The images were acquired using the camera CoolSnapHQ2 (Photometrics, Canada) equipped in TE 2000 microscope (Nikon, Japan). For imaging cytochrome c EGFP, excitation filter of 470±40, emission filter of 525±50 and dichroic 495LP were used. TMRM was viewed through a filter combination of Ex: 545±30, Em: 620±60 and dichroic 570LP. The condensation of chromatin was analysed by staining the cells with 0.5 µg/ml of Hoechst for 10 minutes followed by imaging using the filter combination Ex: 350±50 nm and Em: 460±60 nm. The condensed nuclei with intense fluorescence were counted from the total nuclei in the field to calculate the percentage of cells with condensed nuclei. Similarly for quantitative measurement of cells with caspase activation from ratio images, cells showing saturated donor and acceptor fluorescence were excluded to minimize saturation dependent artifacts

For imaging cells in 96 well plates, cells were seeded in 96 well glass bottom plates (Greiner Bio- One) at desired density. After 24 h, medium was removed and replenished with 5% FBS supplemented phenol-red free DMEM containing the zerumbone at various concentrations. The same XY position was repeatedly imaged using Pathway Bio-imager 435 (Becton Dickinson,USA) at defined time periods as described earlier [12]. Images of cells in each well were acquired in the respective channels using a dry 20X objective with NA 0.75. The excitation/emission filter 350±50 nm/460±60 nm for Hoechst and 530±25 nm/592±22 nm filter for TMRM were coupled along with the ECFP and EYFP FRET filter combination for simultaneous visualization of chromatin condensation and ΔΨ_m_. Image analysis was carried out using software Attovision 1.6/435 (Becton-Dickinson).

## Results

### Zerumbone induced cytotoxicity involved chromatin condensation

**Figure 1:**
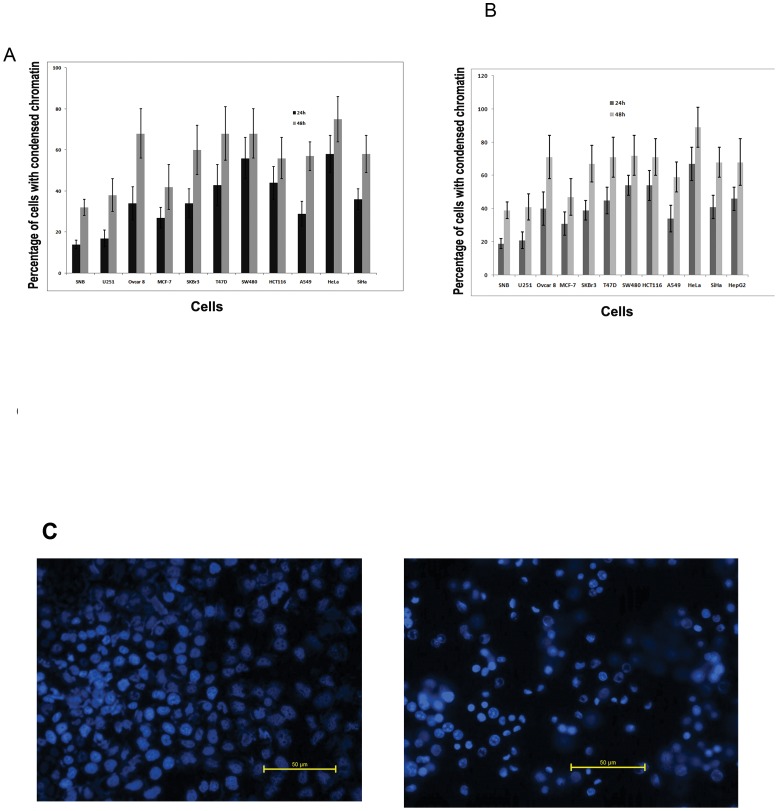
Zerumbone induced cytotoxicity involved chromatin condensation. (A). The indicated cells were treated with zerumbone 50 µM for 24 and 48 h. Then the cells were trypsinized and stained with trypan blue to calculate the percentage positive cells with trypan blue uptake by microscopy. The data shown are average ±SD (n = 4). (B). The indicated cells were grown on 96 well plate and treated with zerumbone 50 µM for 24 and 48 h. Then the cells were stained with Hoechst dye as described. The cells with intense, condensed chromatin were counted from three independent wells to calculate the percentage of cells with condensed chromatin. The results shown are average ± SD (n = 4). (C). Representative fluorescent image showing condensed chromatin after zerumbone 50 µM treatment for 48 h in SKBr3 cells (40x).

A panel of cancer cell lines of different tissue origin was employed to profile cytotoxic activity of zerumbone. Based on the results from the preliminary cytotoxic studies, the cells were incubated with 50 µM of zerumbone for 24 h and 48 h and cell death was analysed by trypan blue staining. As shown in **figure** **1** **A**, zerumbone induced cell death in most of the cell lines in time dependent manner as indicated by increased percentage of cells with trypan blue uptake. The Ovcar8, HeLa, SW480 and T47D showed more sensitivity to zerumbone compared to other cancer cells. The breast cancer cell line MCF-7 and cervical cancer cell line SiHa showed moderate cytotoxicity at 24 h. In general, the neuroblastoma cell lines U251 and SNB19 were least sensitive to zerumbone. However, these cells showed almost 40–50% of cell death at 48 h with 50 µM of zerumbone. In order to address whether the cytotoxic activity is associated with apoptosis, general parameter of apoptosis i.e. condensation of nucleus was analysed in multiple cells using fluorescent microscopy. Consistent with the cytotoxic activity, zerumbone induced chromatin condensation in multiple tumor types in a cell type dependent manner **(** **Figure** **1B**). A representative image of chromatin condensation is also shown in **figure** **1** **C**. Over all, the results substantiate that zerumbone is capable of inducing cell death by apoptosis in tumor types of varied tissue origin.

### Zerumbone induced caspase activation subsequent to mitochondrial transmembrane potential loss (ΔΨ_m_)

**Figure 2:**
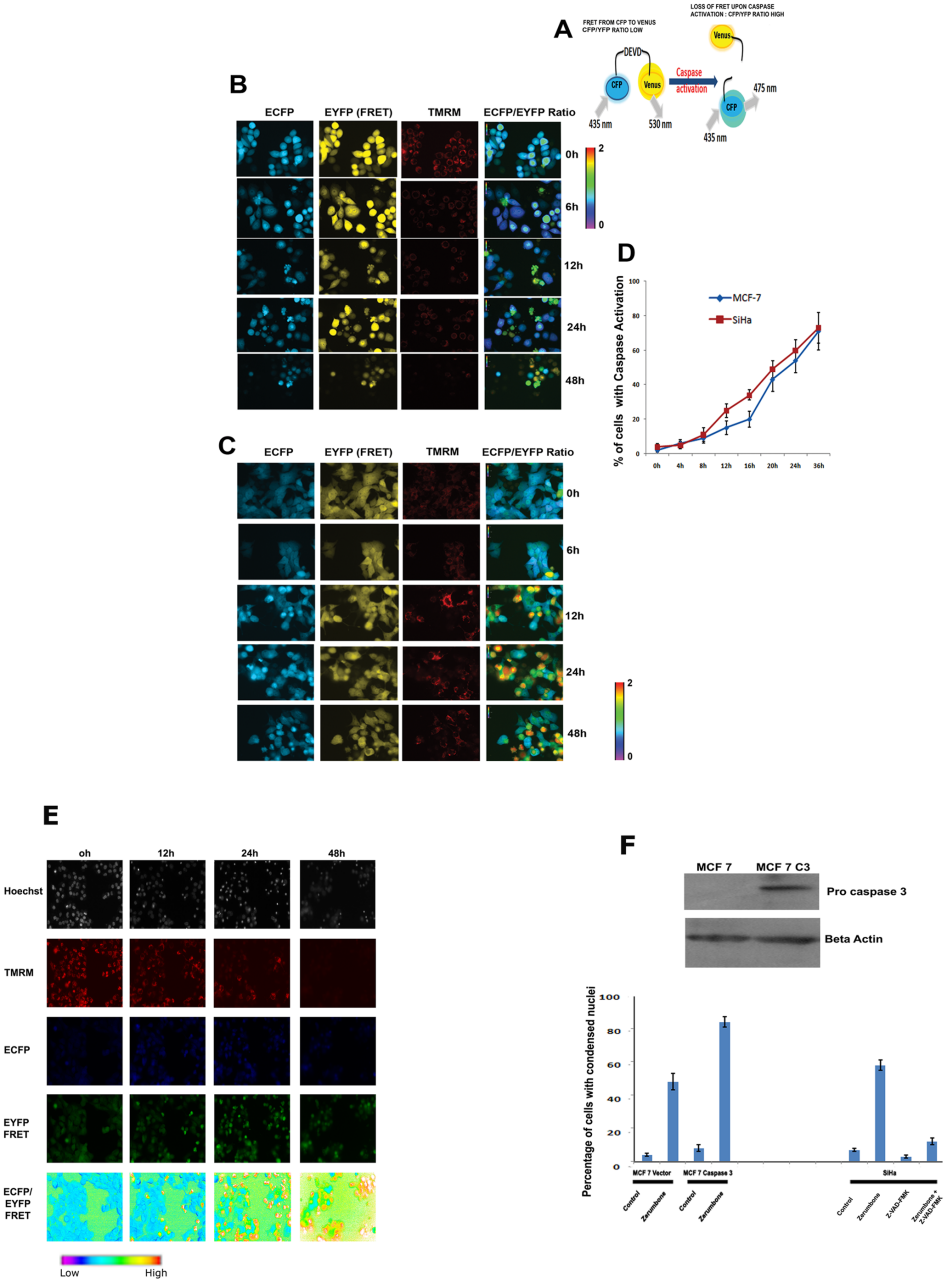
Zerumbone induced caspase activation subsequent to mitochondrial transmembrane potential loss. (A). Schematic representation of live cell caspase probe employed to detect caspase activation. The cells express ECFP-DEVD-VENUS fusion protein. DEVD is the preferred amino acid sequence of activated caspase 3 and caspase 7 that is linked in between the donor fluorophore, ECFP and acceptor fluorophore, EYFP. If caspases are not activated, when ECFP is excited energy is transferred to EYFP because of the FRET between the donor and acceptor pairs leading to decrease in ECFP fluorescence and increase in EYFP fluorescence. Upon caspase activation, DEVD is cleaved with loss of FRET that changes the ratio of ECFP/EYFP. (B). Cervical cancer cell line SiHa expressing ECFP-DEVD-EYFP were stained with 50 nm TMRM for 10 min. Then the cells were treated with zerumbone 50 µM in medium containing 10 nm TMRM. The images were taken at 0h, 6 h, 12 h and 24 h using the filter combinations described in the materials and methods under live cell incubation on stage. ECFP channel, EYFP FRET channel, ratio image and TMRM channels are shown. Caspase activation is reflected in ratio change and loss of ΔΨm in TMRM fluorescence intensity by decrease, diffuse or loss. (C). Breast cancer cell line MCF-7 expressing ECFP-DEVD-EYFP were stained with 50 nm TMRM for 10 min. Then the cells were treated with zerumbone 50 µM in medium containing 10 nm TMRM. The images were taken at 0 h, 6 h, 12 h and 24 h using the filter combinations described in the materials and methods under live cell incubation on stage. ECFP channel, EYFP FRET channel, ratio image and TMRM channels are shown. Caspase activation is reflected in ratio change and loss of ΔΨm in TMRM fluorescence by decrease, diffuse or loss. (D). SiHa cells and MCF-7 cells expressing FRET probe were treated with zerumbone 50 µM and ratio imaging was carried out as described. The cells with FRET loss were calculated and represented as graph (n = 4). (E). Ovcar 8- ECFP- DEVD-EYFP cells were stained with Hoechst and TMRM, treated with Zerumbone 50 µM. Imaging for Hoechst, TMRM, ECFP, and EYFP FRET were carried out using a 96 well plate Bio-imager as described at the indicated time points. The contrasts of ECFP and EYFP FRET images are linearly adjusted for visual purpose. (F). MCF-7 cells were transfected with vector alone or pc DNA3 Caspase 3 vector. The caspase 3 expression was analysed by western blot and shown. The stable clones were treated with zerumbone 50 µM. After 24 h chromatin condensation was analysed as described. The right panel represents SiHa cells treated with zerumbone 50 µM alone or after pretreatment with caspase inhibitor z VAD-FMK (50 µM) followed by zerumbone 50 µM for 24 h. The chromatin condensation data from three independent experiments were used for generating the graph.

Condensation of chromatin is executed by cleavage of structural proteins of nucleus by activated caspases. Further to substantiate caspase activation and to study the kinetics of caspase activation by zerumbone, a sensitive live cell FRET based tool for detecting caspase activation was employed as described [8]. The FRET probe is designed as cyan fluorescent protein (donor) and yellow fluorescent protein (acceptor) which are linked together by activated caspase-3 and caspase-7 cleavable amino acid sequence “DEVD”. Upon activation of caspases within the cells, the linker DEVD sequence gets cleaved, leading to the separation of both fluorophores and resulting in loss of FRET between the probes (**Figure** **2** **A**)**.** Loss of FRET during caspase activation will be reflected as a change in ratio of ECFP/EYFP in ratio imaging platform as described [12]. Initially we have employed cervical cancer cell line SiHa that showed moderate cytotoxicity to zerumbone to study the kinetics of caspase activation using FRET probe expressing cells by microscopy. The cells were also stained with TMRM, a mitochondrial membrane potential specific dye for simultaneous visualization of loss of ΔΨ_m_ and caspase activation. As shown in the **figure** **2** **B**, the untreated cells showed intense mitochondrial red fluorescence indicating intact ΔΨ_m_ and low CFP/YFP ratio indicating the FRET between the CFP and YFP. After 6h of treatment, 6–8% of cells showed increase in ratio indicating that the probe is cleaved by activated caspases. By 12 h almost 22% cells showed change in ratio which was further increased to 56% by 24 h. By 48 h, almost 78% cells were dead by caspase activation. Similarly, significant caspase activation was also noticed in MCF-7 cells after reduction in TMRM intensity (**Figure** **2** **C and D**)**.** Interestingly, all cells with high ratio also showed reduction in TMRM intensity indicating that caspase activation is downstream of ΔΨ_m_ loss. Since few cells showed increased donor and acceptor fluorescence after drug treatment, these cells were excluded while counting cells with FRET loss to avoid fluorescence saturation dependent artifacts. Further live cell imaging for TMRM and ratio was carried out at an interval of 5 minutes from 24 h of drug treatment to understand the time interval from TMRM loss and caspase activation. A representative video of TMRM and corresponding ECFP/EYFP FRET ratio images are provided as supplementary video (Movie S1 and Movie S2). The results suggest that TMRM loss was followed by caspase activation with a time gap of 5–15 minutes in cells undergoing caspase activation and death. Two more cancer cell lines expressing caspase sensor probe were employed to analyze the kinetics of caspase activation in cells treated with zerumbone using a high-throughput imager at defined time points (**Figure** **2** **E and Figure S1**). The cells were also stained with TMRM and Hoechst stain to visualize the ΔΨ_m_ loss and chromatin condensation respectively induced by zerumbone. Consistent with the earlier results of chromatin condensation, neuroblastoma cells showed relative resistance to zerumbone induced caspase activation. Further to substantiate caspase dependency of cell death by zerumbone, SiHa cells were pretreated with cell permeable broad spectrum caspase inhibitor, z-VAD-FMK that significantly reduced the percentage of cell death (**Figure** **2** **F**). Again, ectopic introduction of caspase 3 in caspase 3 deficient MCF-7 cells increased the cell death (**Figure** **2** **F**) emphasizing caspase 3 dependency of zerumbone mediated cell death. Overall, the results indicate that zerumbone induces cell death primarily through caspase dependent manner that is associated with loss of ΔΨ_m_ loss and condensation of chromatin. The simultaneous imaging of ΔΨ_m_ and caspase activation revealed caspase activation was downstream of ΔΨ_m_ loss in zerumbone induced cell death.

### Zerumbone induced cytochrome c release after mitochondrial permeabilisation

**Figure 3:**
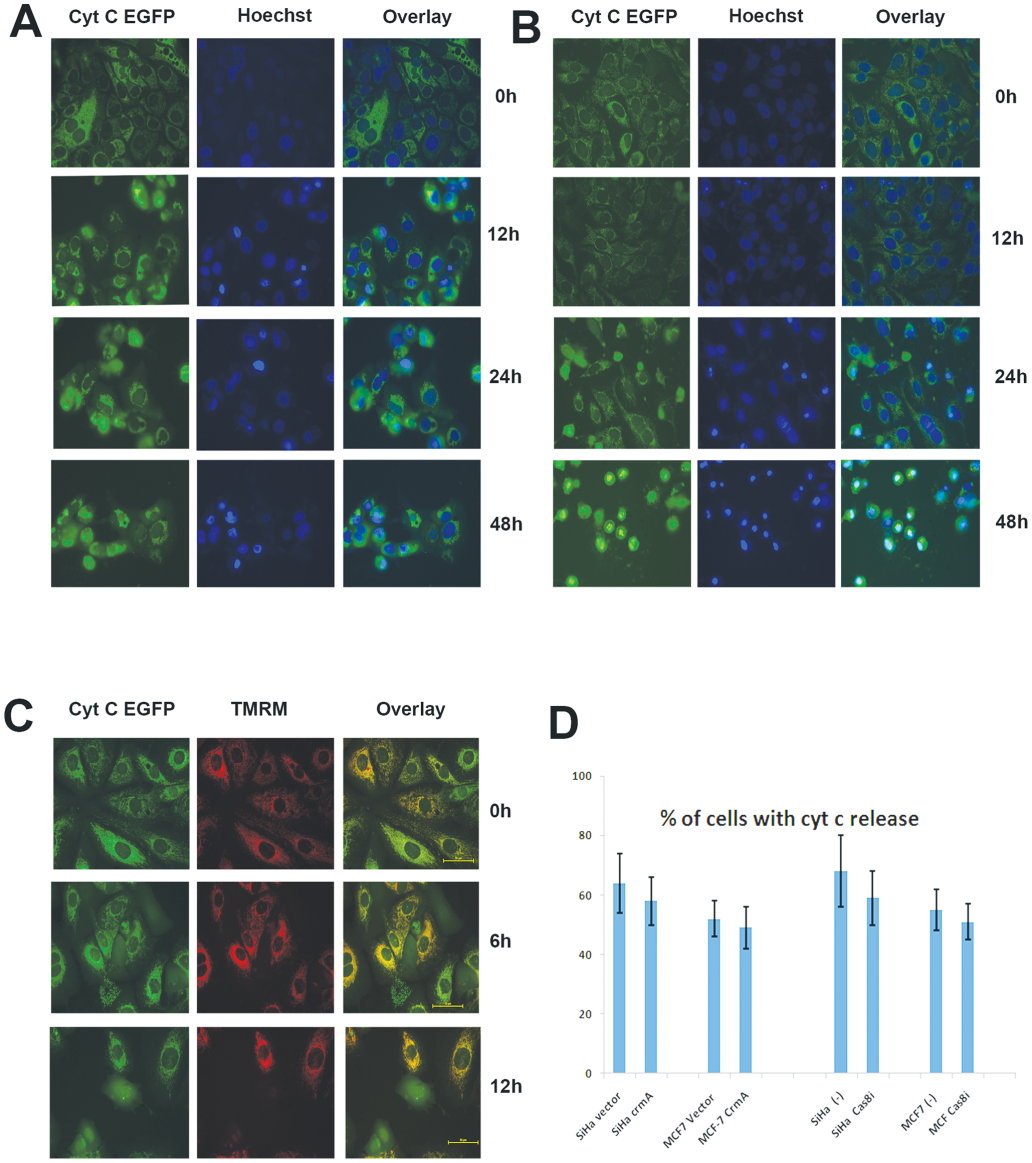
Zerumbone induced cytochrome c release after mitochondrial permeabilisation. (A). MCF-7 cells expressing cytochrome c EGFP were treated with zerumbone 50 µM for indicated time points. The nucleus was stained with Hoechst to visualize chromatin condensation. Imaging was carried out at 0 h, 12 h, 24 h and 48 h as described under live cell incubation on stage. The cells with diffuse cytoplasmic green fluorescence indicate the release of cytochrome c from mitochondria. (B). SiHa cells expressing cytochrome c EGFP were treated with zerumbone 50 µM for indicated time points. The nucleus was stained with Hoechst to visualize chromatin condensation. Imaging was carried out at 0 h, 12 h, 24 h and 48 h as described under live cell incubation on stage. The cells with diffuse cytoplasmic green fluorescence indicate the release of cytochrome c from mitochondria. (C). MCF-7 cells expressing cytochrome c EGFP were stained with 50 nm TMRM and treated with zerumbone 50 µM in 10 nM TMRM containing medium for indicated time points. Imaging for EGFP and TMRM was carried out at 0 h, 6 h and 12 h as described. The cells with diffuse cytoplasmic green fluorescence indicate the release of cytochrome c from mitochondria and loss of TMRM red fluorescence indicate loss of ΔΨm. (D). Cytochrome c EGFP expressing SiHa and MCF-7 cells were transfected with vector alone (pcDNA3) or with pCDNA3 CrmA. 24 h after transfections, cells were treated with zerumbone 50 µM for 24 h. An independent experiment was done with cells pretreated with 30 µM of caspase8 inhibitor (z-IETD-FMK). The cells with diffuse cytochrome c were counted from three different fields to calculate percent positive cells with diffuse cytoplasmic cytochrome c. The results shown are average + SD from four independent experiments (n = 4)

Two independent pathways generally categorized as extrinsic and intrinsic pathways contribute for caspase activation in mammalian cells. The intrinsic apoptosis signaling is initiated with limited permeabilisation of mitochondria leading to the release of cytochrome c in to cytosol that triggers caspase dependent cell death through activation of procaspase 9 in the apoptosome complex. In order to analyze the role of mitochondria and cytochrome c dependency, we have used SiHa and MCF-7 cells expressing cytochrome c-EGFP fusion protein as described earlier [13]. As shown in **figure 3** **A and B,** after 12 h of treatment with zerumbone (50 µM), cytochrome c release was initiated in few cells with a time dependent increase (**Figure** **3** **B**). Simultaneous staining of cells with Hoechst reflected condensed chromatin in cells showing diffused cytochrome c. Furthermore, simultaneous imaging of cytochrome c release and TMRM indicated that ΔΨ_m_ loss precedes cytochrome c release in zerumbone induced apoptosis indicating for a classical intrinsic death signaling (**Figure** **3** **C**). However, both intrinsic as well as extrinsic pathways of apoptosis are known to contribute for cytochrome c release subsequent to loss of ΔΨ_m_. During extrinsic pathway, activated caspase 8 cleaves cytosolic Bid to generate tBid which triggers mitochondrial membrane permeabilisation by stimulating proapoptotic Bax and Bak proteins at mitochondria. However, activated caspase-3 as well as other caspases are known to cleave Bid. So to define the signaling, we have used a caspase-8 inhibitor as well as cells transfected with CrmA that specifically inhibit caspase 8 processing. As shown in **figure** **3** **D**, both caspase 8 inhibitor as well as CrmA failed to inhibit cytochrome c release induced by zerumbone indicating that cytochrome c release was evoked primarily by intrinsic pathway of apoptosis.

### Zerumbone induced apoptosis requires Bax

**Figure 4:**
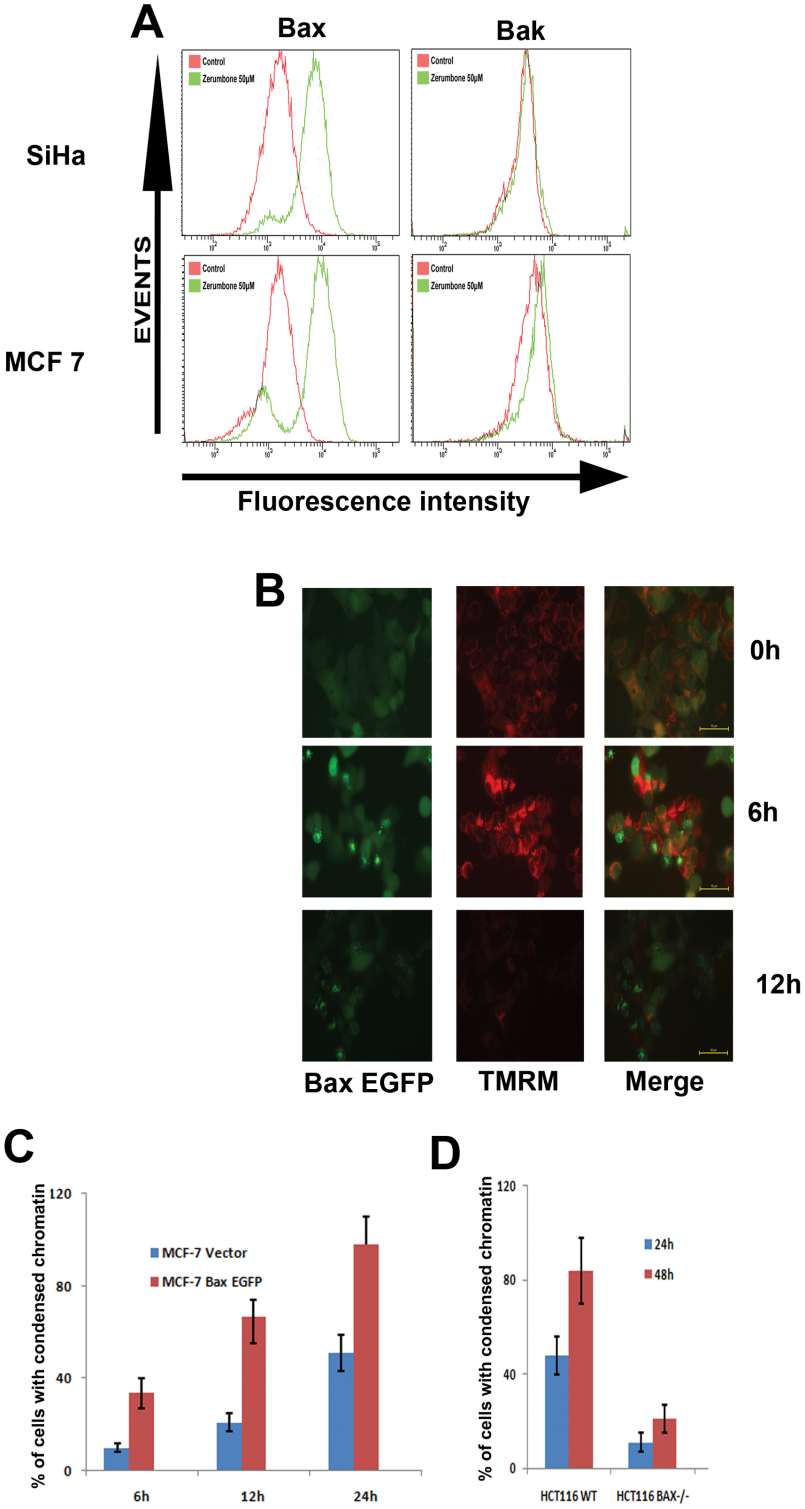
Zerumbone induced apoptosis requires Bax. (A). SiHa and MCF-7 cells were treated with zerumbone 50 µM for 24 h. Then the cells were fixed permeabilised and immunostained for conformationally active Bax (6A7) or conformationally active Bak as described. Analysis was carried out using FACSAria to determine fluorescence intensity. (B). MCF-7 cells expressing Bax EGFP were stained with 50 nm TMRM and treated with zerumbone 50 µM in 10 nM TMRM containing medium for indicated time points. Imaging for EGFP and TMRM was carried out at 0 h, 6 h and 12 h under live cell incubation on stage. The cells with granular perinuclear green fluorescence indicate Bax oligomerisation at mitochondria and loss of TMRM red fluorescence indicate ΔΨm loss. (C). MCF-7 cells expressing Bax EGFP and vector control transfected cells were treated with zerumbone 50 µM for 12 and 24 h and analysed for chromatin condensation. (D). Colon cancer cell line HCT116 and its Bax deficient derivative Bax KO was treated with zerumbone 50 µM for 24 h and 48 h and analysed for chromatin condensation.

The above results indicate that zerumbone is capable of inducing cell death through classical apoptosis where mitochondria essentially act as initiator of the death cascade. Mitochondrial permeabilisation, the early event required for cytochrome c release from mitochondria is tightly regulated by anti-and pro-apoptotic Bcl 2 family proteins. Conformational activation of proapoptotic Bax or Bak targets mitochondrial outer membrane which leads to its permeabilisation and cytochrome c release. We have employed specific antibodies to detect activated form of Bax and Bak by flow cytometry. As shown in **figure** **4** **A,** compared to untreated control, an enhanced level of conformational active Bax was detected in both the cell lines by 50 µM of zerumbone treatment. A mild activated Bak was also observed in zerumbone treated MCF-7 cells. Consistent with this, zerumbone treated cells showed increased expression of conformational active Bax by immuno fluorescent detection (Data not shown). To substantiate the role of Bax, we have expressed Bax-EGFP in MCF-7 cell lines and kinetics of Bax activation was evaluated after treatment with zerumbone by live cell time-lapse imaging. As shown in **figure** **4** **B**, 6 h of zerumbone treatment enhanced Bax activation and its translocation to mitochondria in 27% of cells which was further increased to 90% by 24 h. Consistent with the proapoptotic activity of Bax, Bax overexpression sensitized the cells and enhanced the cell death as evident from Bax translocation and chromatin condensation (**Figure** **4** **B &C**)**.** Simultaneous study of Bax-EGFP and mitochondrial membrane potential enabled us to substantiate that loss of TMRM fluorescence is immediately followed by formation of punctuated Bax-EGFP in most of the cells indicating that ΔΨ_m_ loss and subsequent Bax conformational activation are necessary for the cell death induced by zerumbone. Bax activation and Bax translocation to mitochondria are temporally distinct events; only massively oliogomerized Bax at mitochondria is visible as aggregates. The results suggest that Bax activation contributes to immediate ΔΨ_m_loss.

Further to substantiate the importance of Bax in zerumbone induced cell death, we have employed isogenic cell types with Bax deficiency. The colon cancer cells HCT116 Bax knock out variant is a Bax deficient cells generated with homologous recombination technology [7]. As shown in **figure** **4** **D**, deficiency of Bax, significantly reduced the percentage of cells with condensed chromatin compared to wild type cells.

### Calcium dependent calpain and ROS contribute for Bax activation and mitochondrial permeabilization

**Figure 5:**
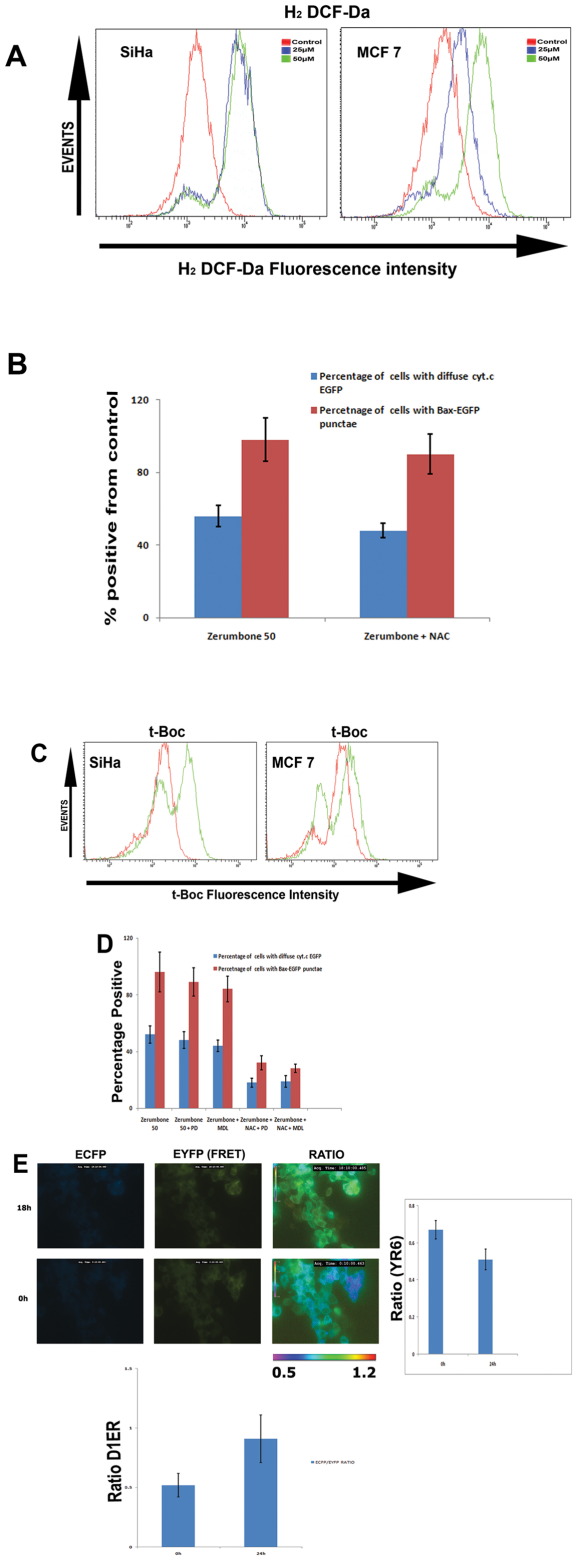
Calcium dependent calpain and ROS contribute for Bax activation and mitochondrial permeabilization. (A). SiHa and MCF-7 cells were treated with zerumbone 25 µM and 50 µM for 24 h. Then the cells were trypsinized and loaded with DCF-DA (10 μM) in serum free OptiMEM medium and immediately analysed by flow cytometry (FACSAria). (B). MCF-7 cells expressing cytochrome c EGFP and Bax EGFP were pretreated with N Acetyl Cysteine (NAC) (3 mM) or DMSO alone for 1 h followed by zerumbone treatment for 24 h. The cells with diffuse cytochrome and Bax punctae were counted and represented as graph. Values are average ± SD from four different experiments. (C). SiHa and MCF-7 cells were treated with zerumbone 50 μM for 24 h. Then the cells were trypsinised and stained with 10 μM t-BOC as described. The fluorescence was analysed using flow cytometry. (D). MCF-7 cells expressing cytochrome c EGFP and Bax EGFP were pretreated with indicated inhibitors alone and inhibitors for 1 h followed by zerumbone for additional 24 h. The cells with diffuse cytochrome and Bax punctae were counted and represented as graph. Values are average + SD from four different experiments. (E). MCF-7 cells expressing calcium probe chameleon targeted at ER (D1ER) was treated with zerumbone for 12 h. The ECFP-EYFP ratio imaging was carried out as described under live cell incubation on stage at an interval of 5 minutes from 12 h onwards. The average ratios from healthy cells and rounded dead cells at 24 h were calculated using NIS element software and plotted. The cells expressing chameleon at cytoplasm was also treated in the same manner to perform ratio imaging. The average ratio is shown as graph (n = 4). (**p ≤001). The contrasts of ECFP and EYFP FRET images are linearly adjusted for visual purpose.

The results shown above indicate activation of Bax and its essential role as an early event of cell death in zerumbone treated cells. Even though the precise mechanism of Bax conformational activation during apoptosis is still elusive; the potential role of reactive oxygen species in Bax activation has been indicated in multiple cell death signaling [14], [4]. We have analysed the ROS levels after treating the cells with zerumbone 25 and 50 µM for 24 h. As shown in **figure** **5** **A,** zerumbone significantly increased fraction of cells with high ROS in both the cells. Since Bax activation and cytochrome c release were the early events induced by zerumbone, we further evaluated whether these two events were affected in cells pretreated with ROS scavengers, NAC. As shown in **figure** **5** **B**, pretreatment of cells with NAC failed to prevent Bax activation and cytochrome c release indicating ROS independent triggers for Bax activation. Activated calpains are known to activate Bax in apoptotic cell death [15]. Calpain activity was analysed using calpain specific fluorescent probe t-BOC by FACS that showed an increased population with enhanced calpain activity in zerumbone treated cells (**Figure** **5** **C**).

Consistent with this pretreatment of both NAC and calpain inhibitors (PD and MDL) significantly prevented Bax activation and cytochrome c release. However pretreatment of only calpain inhibitor showed moderate inhibition of cell death (**Figure** **5** **D**). Again increase in intracellular calcium and release of ER calcium by zerumbone was evaluated using MCF-7 cells expressing Chameleon probe at cytosol and ER. Our initial studies using FURA2 stained cells failed to show any immediate calcium release in zerumbone treated cells. So we have analysed the calcium change after 24 h of zerumbone treatment, a time point where apoptotic changes are visible. As shown in the **figure** **5E** by 24 h of zerumbone treatment an increase in D1ER fluorescence ratio (ECFP/EYFP) was observed compared to 0h indicating the release of Endoplasmic reticulum calcium. The time dependent change in ratio is shown as supplementary video. (Movie S3). As shown in the video, increase in ratio is evident in all cells before cell rounding. Consistent with this, a decrease in fluorescence ratio for cytosolic chameleon was observed reflecting the corresponding increase in cytosolic calcium. The results indicate that calcium dependent calpains as well as ROS collectively contribute to Bax activation leading to cytochrome c release from mitochondria where ER calcium release play a key role.

### ER targeted Bcl 2 prevents cell death induced by zerumbone than wild type Bcl 2 or Bcl-XL

**Figure 6:**
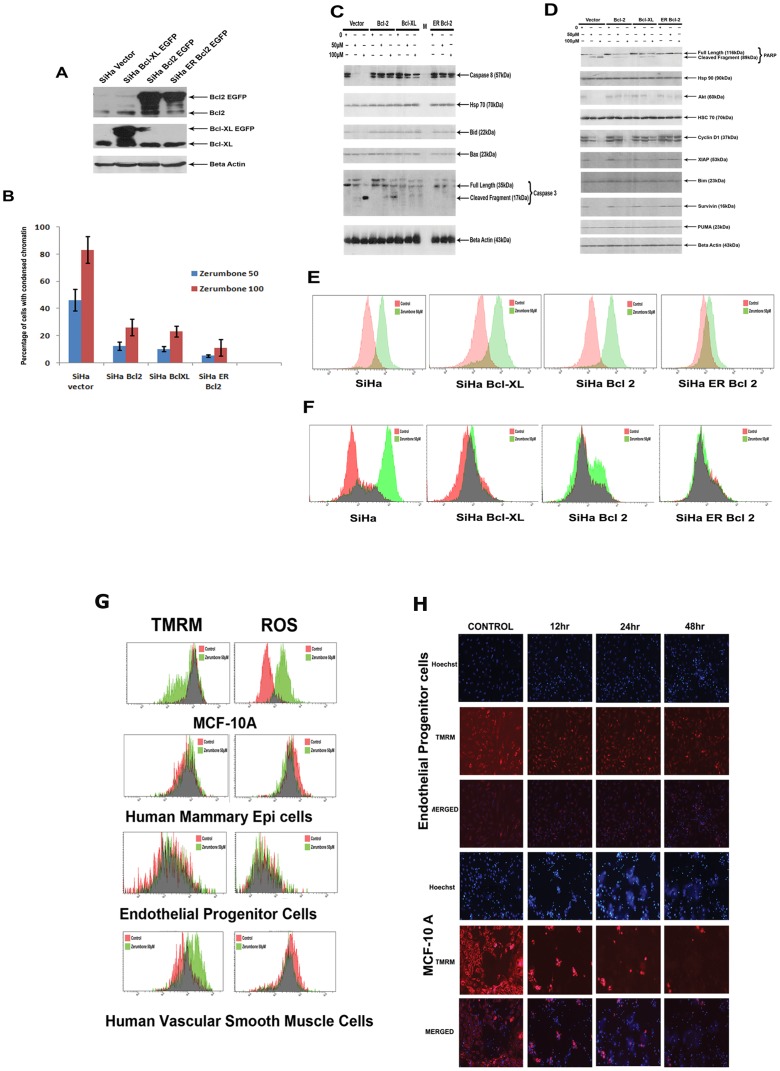
ER targeted Bcl 2 prevents cell death induced by zerumbone than wild type Bcl 2 or Bcl-XL. A. SiHa cells were transfected with vector alone or Bcl-XL –EGFP, Bcl2- EGFP and ER-Bcl2. The whole cell extract prepared from the cell was probed for Bcl2 and Bcl-XL. Beta actin is served as the loading control. (B). The above panel cell lines were treated with zerumbone 50 µM and 100 µM for 24 h. Then the cells were stained with Hoechst to quantify chromatin condensation. The results shown is average ±SD (n = 4)(**p≤001). (C). The whole cell extract prepared from vector alone or BclXL –EGFP, Bcl2 EGFP and ERBcl2, untreated, or treated with zerumbone 50 µM and 100 µM for 24 h were probed with antibodies against caspase 8, hsp70, Bid, Bax, caspase 3 by western blot technique. Beta actin served as loading control. (D). The whole cell extract prepared from vector alone or Bcl-XL –EGFP, Bcl2 EGFP and ER-Bcl2, untreated, or treated with zerumbone 50 µM and 100 µM for 24 h were probed with antibodies against hsp90, Akt, cyclin D1, XIAP, Survivin, PUMA, by westernblot technique. β-actin and hsc70 served as loading control. (E). SiHa vector alone, BclXL –EGFP, Bcl 2 EGFP and ERBcl2, untreated, or treated with zerumbone 50 µM were stained with Cell ROX Red as described and analysed by flow cytometer. (F). SiHa vector alone, BclXL –EGFP, Bcl 2 EGFP and ERBcl2, untreated, or treated with zerumbone 50 µM were stained with t-BOC as described and analysed by flow cytometer. (G). MCF-10 A, Human Mammary epithelial cells, human smooth Muscle cells and endothelial progenitor cells were treated with zerumbone 50 µM for 24 h. Then the cells were stained with TMRM or DCF-DA as described and analysed by flow cytometer. (H). Endothelial progenitor cells and MCF10A cells were stained with TMRM and Hoechst followed by zerumbone50 μM treatment. The wells were repeatedly imaged at the indicated time points.

The results shown above confirmed the importance of mitochondria and Bax activation in mediating the cell death by zerumbone and identified ROS and calpain as the critical initiators. In mammalian cells, Bcl 2 family proteins acts as the gate keepers of cell death at mitochondria and plays a critical role in regulating Bax induced mitochondrial permeabilisation. Consistent with this knowledge, overexpression of anti-apoptotic proteins like Bcl 2 and Bcl-XL often renders cancers non responsive to drugs and remains as the major cause for clinical drug failure. Since our study identified independent role for ROS and calpain in targeting Bax, further studies were done to address whether the apoptosis induced by this compound is influenced by antiapoptotic Bcl 2 family proteins. SiHa cells stably expressing Bcl 2, Bcl-XL or Bcl 2 targeted at ER were generated as described (**Figure** **6** **A**)**.** These cells showed marked resistance against most of the apoptosis inducing stimuli than the vector control cells (Data not shown). These cells were exposed to 50 µM and 100 µM of zerumbone for 24 h and cell death was analysed by chromatin condensation as described. As shown in the figure (**Figure** **6** **B**) compared to vector control cells Bcl 2 and BclXL expressing cells were significantly resistant to zerumbone treatment. Interestingly cells expressing Bcl 2 at ER were more protected than the wild type Bcl 2 cells suggesting a prominent role of ER calcium and calpain activation in zerumbone induced cell death. Western blot data showed caspase8 activation in treated vector transfected cells that was reduced in both Bcl 2 and Bcl-XL over expressing clones (**Figure** **6** **C**)**.** Caspase 3 activation was also reduced in Bcl2 and Bcl-XL overexpressing cells. The enhanced protection of ER Bcl 2 was reflected in PARP cleavage also.

In an effort to identify other key regulators of cell death in zerumbone induced apoptosis, expression of the proapoptotic proteins like PUMA, BIM ,BAX and survival proteins like Survivin, XIAP, heat shock proteins and cyclin D1 were evaluated in the panels of cells. The key anti- apoptotic protein, XIAP, Survivin and Akt were down regulated in zerumbone treated cells where as both Bcl2 and Bcl-XL prevented their down regulation. Hsp90 and hsp70 were not affected in zerumbone treated cells. Interestingly the downregulation of Survivin and XIAP induced by zerumbone was completely prevented only in cells expressing Bcl 2 at ER (**Figure** **6** **D**)**.** Since we have observed enhanced protection in ER Bcl 2 expressing cells, analysis of ROS and calpain activity was carried out in Bcl 2 expressing clones. Consistent with the above data, zerumbone failed to generate both ROS and active calpain in ER Bcl 2 expressing cells compared to Bcl 2 or Bcl-XL over expressing cells (**Figure** **6** **E and F**).

Among natural products, zerumbone received special attention because of its selective toxicity towards cancer cells compared to the normal cells. In the initial studies only few normal cells were employed. We have extended this study to profile its activity in additional and physiologically relevant normal diploid proliferating cells. The results presented in this section reveals that Bcl 2 family proteins like Bcl 2 and Bcl-XL prevent cell death induced by zerumbone and more protection is afforded when Bcl 2 was expressed at endoplasmic reticulum again substantiating the important role of ER calcium and calcium dependent calpain in mediating the cell death in cooperation with ROS (**Figure** **6** **E and F**). Again the results supports a redox mediated diverging role for its selectivity to cancer cells sparing proliferating diploid cells. Since intracellular generation of ROS and ΔΨm loss are the two important triggers and apoptotic events in zerumbone induced cell death, both these events were analysed in the treated normal cells also. As shown in the **figure** **6** **G and H** endothelial progenitor cells, mammary epithelial cells, smooth muscle cells were resistant to ΔΨ_m_ loss induced by zerumbone, however MCF10A remained sensitive to zerumbone. Interestingly the normal diploid cells that showed resistance to zerumbone showed less intracellular ROS upon treatment. In fact some of the resistant diploid cells showed increased transmembrane potential upon zerumbone treatment (**Figure** **6** **H**). Consistent with this calpain activation was also negligible in normal cells treated with zerumbone (Figure S2).

## Discussion

Plants and natural products remain as the ideal resource in search for drug discovery because of their unique structural diversity and promising long term safety records. Despite their extensive use for various medicinal applications, only few compounds reached clinic as successful antitumor agents. Among large number of natural products evaluated as experimental drugs against cancer, zerumbone received special attention because of its selective toxicity towards cancer cells compared to the normal cells. Xiang Mingji et al reported that zerumbone is capable of inducing cell death in leukemic cells via FAS receptor activation, Bid cleavage and caspase 3 activation but not in normal endothelial cells [2]. Previously, studies by Murakami etal showed that zerumbone is non toxic to normal cells however inhibited proliferation and induced cell death in colon cancer cell [16]. A promising hypothesis involving intracellular redox regulated mechanisms was proposed to account for the selectivity of zerumbone [17].

The wide variety of biological activity reported for zerumbone include metastasis inhibition through chemokine receptor CXCR4 downregulation, intracellular generation of ROS, induction of pro inflammatory cytokines, inhibition of NFkappa B and IkappaB and its targets [3], [4], [5]. Zerumbone is also a potent inhibitor of inflammation and suppresses skin tumor initiation in mice as well as azoxymethane induced abnormal crypt formation in rats. [18]. An earlier study even though reported its ability to alter Bcl 2 and Bax ratio in treated cells indicating for a mitochondrial death signaling, the precise cellular events that culminates in apoptotic cell death is yet to be defined [19]. Even though necrosis, autophagy and necroapotosis are rarely triggered by certain groups of compounds, the most common route of cell elimination by chemotherapeutics involves induction of apoptosis. Both intrinsic and extrinsic apoptotic signaling are known to be activated in drug treated cells. In the intrinsic death signaling, the compounds primarily target the mitochondria leading to its permeabilisation and release of cytochorme c followed by caspase activation. The early trigger of mitochondrial permeabilisation is conformational activation of the proapoptotic proteins like Bax or Bak that is brought about by a mechanism that still remains as a mystery [20].

The conventional methods of apoptosis detection are end stage assays so is difficult to address hierarchical events of programmed cell death. They are also not suitable for simultaneous analysis of multiple events of cell death with temporal resolution. This is very important in defining apoptosis signaling because of the existence multiple feedback regulation as well as the rapid progression of multiple events once initiated. Hence we employed cancer cells expressing apoptosis sensors to substantiate the events of apoptosis induced by zerumbone with high temporal resolution. We show here that the classical intrinsic apoptosis is triggered in multiple cancer cells that involve mitochondrial permeabilisation and cytochrome c release leading to caspase dependent cell death. Among the proapoptotic proteins that contribute for mitochondrial permeabilisation like Bax and Bak, Bax is specifically activated in zerumbone treated cells. The study using inhibitors enables us to identify ROS as well as calpains as the contributing factors for Bax activation in zerumbone induced cell death. Despite extensive efforts, the molecular switch that governs Bax activation during apoptosis still remains to be determined. An essential role for Bid, BID and PUMA for the activation of Bax and Bax was reported [21]. A role of ROS for Bax activation was also reported in multiple cell death models [22], [23], [24]. Consistent with previous reports, an increased intracellular ROS was noticed in zerumbone treated cells but NAC treatment failed to prevent bax activation and cytochorme c release by zerumbone. However, pretreatment of cells with NAC and calpain inhibitors completely prevented Bax activation and cytochorme c release indicating that both cooperate for Bax activation. Consistent with this we have observed increased activated calpain as well as increased cytosolic calcium in dying cells treated with zerumbone. A moderate delayed ER calcium release was also observed in zerumbone treated cells indicating that calcium dependent calpain as well as ROS contribute for Bax activation that ultimately lead to caspase activation. Overexpression of Bcl 2 family proteins like Bcl 2 and Bcl-XL in general prevent Bax dependent cell death and remains as a major factor for cell death inhibitions [25], [26]. Both Bcl 2 and Bcl-XL overexpression significantly reduced cell death induced by zerumbone; however cells expressing Bcl 2 at ER completely prevented cell death. This again indicates for the prominent role of ER calcium and calpain activity in zerumbone induced cell death. A previous study indicates that ER Bcl2 prevents ER calcium release [27]. Our studies also identified key survival proteins that are down regulated during zerumbone induced cell death. Even though the proapoptotic proteins like PUMA, Bim remains unaffected, Akt, Survivin, XIAP were significantly down regulated in zerumbone treated cells. This is in agreement with a previous study that showed down regulation of NFkappa B target genes in zerumbone treated cells [3], [28]. It is possible that in parallel with its activity on NFkappaB, active calpains may targets XIAP and Survivin that augments post cytochrome c release caspase cascade. This is supported by the observation that in ER Bcl 2 expressing cells both survivin and XIAP remains unaffected compared to Bcl 2 and Bcl-XL expressing cells. Earlier studies indicated that calpains can target and cleave XIAP in *in vitro* systems [29]. A role for death receptor mediated potentiation of TRAIL induced cell death was reported for zerumbone that involves upregulation of death receptor 4 and death receptor 5 [4]. Even though caspase 8 activation was observed in zerumbone treated cells, it is unlikely to be the initiator since CrmA transfections as well as caspase 8 inhibitor failed to inhibit Bax activation or cytochrome c release. Moreover cytochrome c release was observed in caspase 8 deficient neuroblastoma cells IMR32 (Data not shown).

An interesting observation of this study is that several normal cells of varying tissue origin showed variable level of sensititivity to zerumbone. In general endothelial cells, smooth muscle cells and mammary epithelial cells were resistant to zerumbone induced Ψm loss compared to normal fibroblasts' and MCF-7 10A. Most of the resistant cells failed to generate high enough ROS with zerumbone indicating that redox status of the cells plays a key role in determining their sensitivity to zerumbone. This again supports the hypothesis put forwarded by Hoffman et al [17]. Similarly a very recent study by Lekshmi et al identified piperlongumine as cancer selective drug that decreased reduced glutathione to oxidized glutathione in cancer cells but not in normal cells [1]. Further studies in this field are very much essential to identify the critical regulators that are differently expressed in normal sensitive diploid cells and resistant diploid cells against the cancer cells. Currently it is not clear whether the master regulator of antioxidant response Nrf2 plays a decisive role in conferring selectivity. However, contrary to this an earlier study reported that Zerumbone is capable for inducing Nrf2 activity providing a mechanistic explanation for its chemo preventive activity [30], [31]. The results presented here also emphasize the potential applications of live cell probes expressing cells to define the complex apoptosis signaling induced by drug candidates and their ability to track the critical initiating events and the progression of downstream events including caspase activation in real time.

## Supporting Information

Figure S1 **U251 ECFP- DEVD-EYFP cells were stained with Hoechst and TMRM, treated with Zerumbone 50 µM.** Imaging for Hoechst, TMRM, ECFP, and EYFP FRET were carried out using a 96 well plate Bio-imager as described at the indicated time points.
(TIF)

Figure S2 **MCF-10 A, Human Mammary epithelial cells, Human Umbilical Cord Endothelial Cells and endothelial progenitor cells were treated with zerumbone 50 µM for 24 h.** Then the cells were stained with t-BOC as described and analysed by flow cytometer.
(TIF)

Movie S1 **Ovcar 8 DEVD cells were stained with TMRM, treated with zerumbone 50 µM.** Live cell imaging was performed on stage incubator after 24 h of drug treatment at an interval of 5 minutes. TMRM loss or diffusion indicates loss of ΔΨ_m._
(MPG)

Movie S2 **The ECFP/EYFP FRET ratio image of Ovcar 8 DEVD cells from the above experiments described for Movie S1 is shown.** Caspase activation is indicated by increase in ratio.
(MPG)

Movie S3 **MCF-7 cells expressing calcium probe chameleon targeted at ER (D1ER) was treated with zerumbone for 12 h.** After 12 h the ECFP-EYFP ratio imaging was carried out as described under live cell incubation on stage at an interval of 5 minutes. The ratio scale is also shown in the frames.
(MPG)

Table S1 **List of antibodies and its respective dilutions.**
(DOCX)
