# Correction: Calpain and Reactive Oxygen Species Targets Bax for Mitochondrial Permeabilisation and Caspase Activation in Zerumbone Induced Apoptosis

**DOI:** 10.1371/journal.pone.0273729

**Published:** 2022-08-23

**Authors:** Praveen K. Sobhan, Mahendra Seervi, Lokesh Deb, Saneesh Varghese, Anjana Soman, Jeena Joseph, Krupa Ann Mathew, Godi Raghu, George Thomas, E. Sreekumar, S. Manjula, T. R. Santosh Kumar

The 12hr panels for EPC cells in Fig 6H [1] are incorrect as the 24hr EPC panels were duplicated in error. The correct 12hr panels are included in the updated Fig 6 provided with this notice. The raw images underlying the EPC panels in Fig 6H are provided in S1 File.

There appear to be similarities between the 12hr, 24hr and 48hr Hoechst panels for MCF-10A cells in Fig 6H. These images present the same sample and field of view and were captured by live-cell imaging over a 48hr period, and therefore, similarities between the timepoints are expected. The raw images underlying the MCF-10A panels in Fig 6H are provided in S1 File. A detailed description of the method used to capture the images in Fig 6H is provided in S2 File. The *PLOS ONE* editors have no concerns about the MCF-10A panels in Fig 6H.

Raw data underlying all the results reported in the article are available from the corresponding author, except for FCS files underlying the flow cytometry experiments presented in Figs 4, 5, and 6, and Figure S2, which are no longer available. Additionally, the raw image data for the western blots presented in Fig 6C and 6D do not show the full blot area as the membranes were cut prior to staining with antibodies. Please see the correct Fig 6 here.

**Fig 6:**
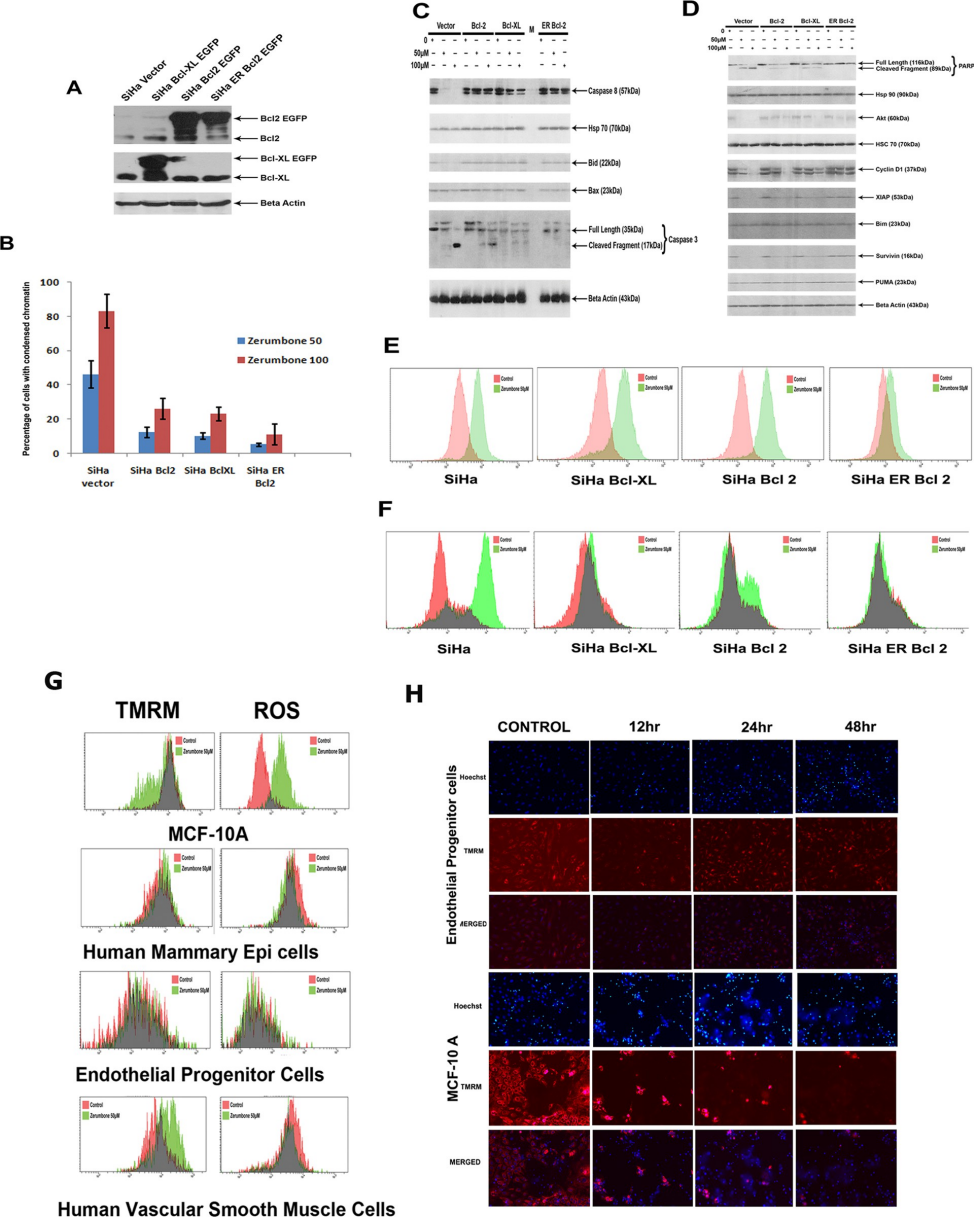
ER targeted Bcl 2 prevents cell death induced by zerumbone than wild type Bcl 2 or Bcl-XL. A. SiHa cells were transfected with vector alone or Bcl-XL–EGFP, Bcl2- EGFP and ER-Bcl2. The whole cell extract prepared from the cell was probed for Bcl2 and Bcl-XL. Beta actin is served as the loading control. (B). The above panel cell lines were treated with zerumbone 50 μM and 100 μM for 24 h. Then the cells were stained with Hoechst to quantify chromatin condensation. The results shown is average ±SD (n = 4)(**p≤001). (C). The whole cell extract prepared from vector alone or BclXL–EGFP, Bcl2 EGFP and ERBcl2, untreated, or treated with zerumbone 50 μM and 100 μM for 24 h were probed with antibodies against caspase 8, hsp70, Bid, Bax, caspase 3 by western blot technique. Beta actin served as loading control. (D). The whole cell extract prepared from vector alone or Bcl-XL–EGFP, Bcl2 EGFP and ER-Bcl2, untreated, or treated with zerumbone 50 μM and 100 μM for 24 h were probed with antibodies against hsp90, Akt, cyclin D1, XIAP, Survivin, PUMA, by western blot technique. β-actin and hsc70 served as loading control. (E). SiHa vector alone, BclXL–EGFP, Bcl 2 EGFP and ERBcl2, untreated, or treated with zerumbone 50 μM were stained with Cell ROX Red as described and analysed by flow cytometer. (F). SiHa vector alone, BclXL–EGFP, Bcl 2 EGFP and ERBcl2, untreated, or treated with zerumbone 50 μM were stained with t-BOC as described and analysed by flow cytometer. (G). MCF-10 A, Human Mammary epithelial cells, human smooth Muscle cells and endothelial progenitor cells were treated with zerumbone 50 μM for 24 h. Then the cells were stained with TMRM or DCF-DA as described and analysed by flow cytometer. (H). Endothelial progenitor cells and MCF10A cells were stained with TMRM and Hoechst followed by zerumbone50 μM treatment. The wells were repeatedly imaged at the indicated time points.

## Supporting information

S1 File Raw image data for Fig 6H. Images were captured using BD Pathway Bio-imager 435 and each file is a composite of four separate images captured as a 2x2 montage.
(ZIP)

S2 File Live-cell imaging method for Fig 6H. (DOCX)
