# Supplementary figures and images for: Calpain and Reactive Oxygen Species Targets Bax for Mitochondrial Permeabilisation and Caspase Activation in Zerumbone Induced Apoptosis
Source: PLoS One. 2013 Apr 9;8(4):e59350. doi: 10.1371/journal.pone.0059350 (PMC3621898; doi:10.1371/journal.pone.0059350)

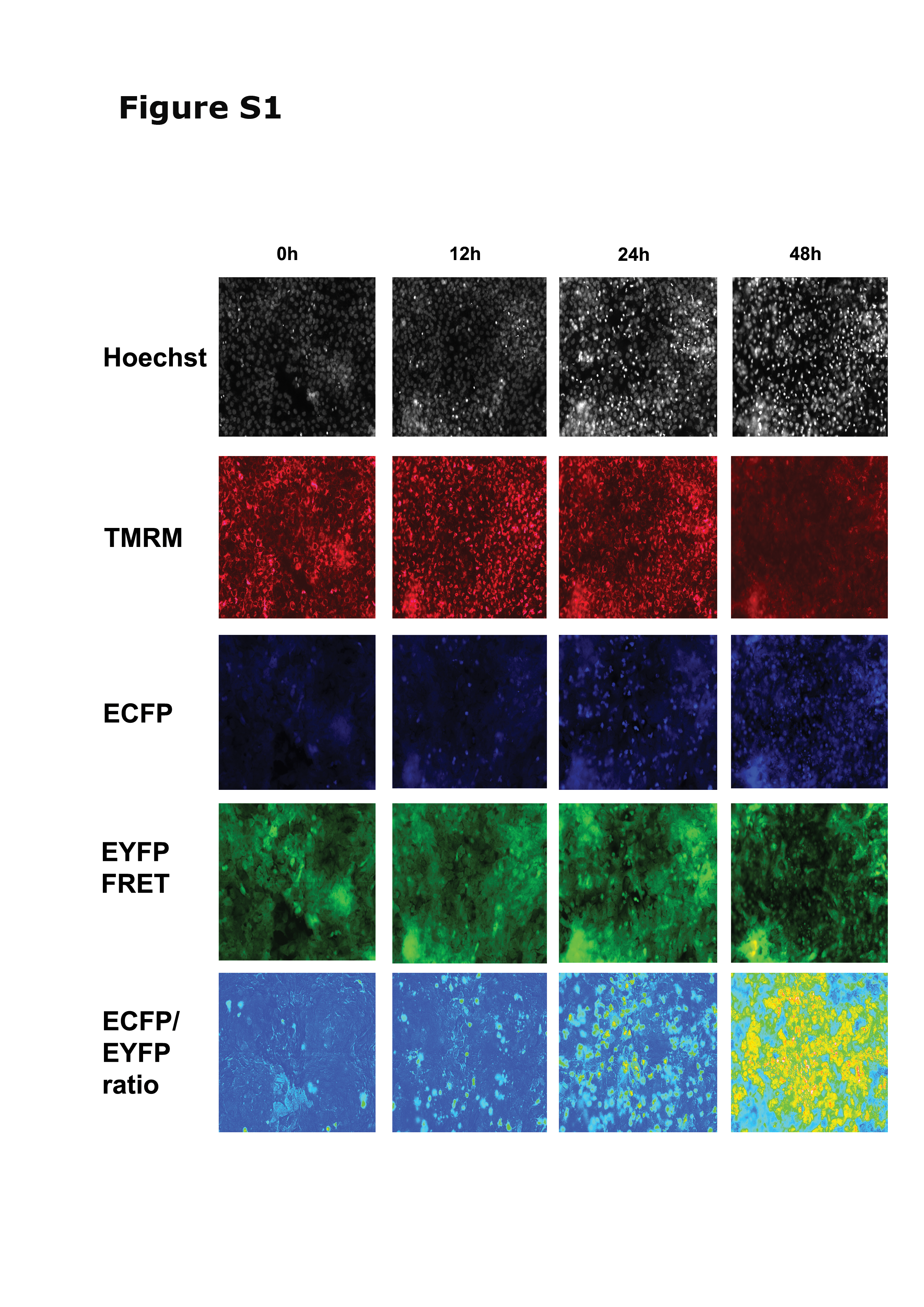

Supplement: Figure S1 — U251 ECFP- DEVD-EYFP cells were stained with Hoechst and TMRM, treated with Zerumbone 50 µM. Imaging for Hoechst, TMRM, ECFP, and EYFP FRET were carried out using a 96 well plate Bio-imager as described at the indicated time points. (TIF) [file pone.0059350.s001.tif]

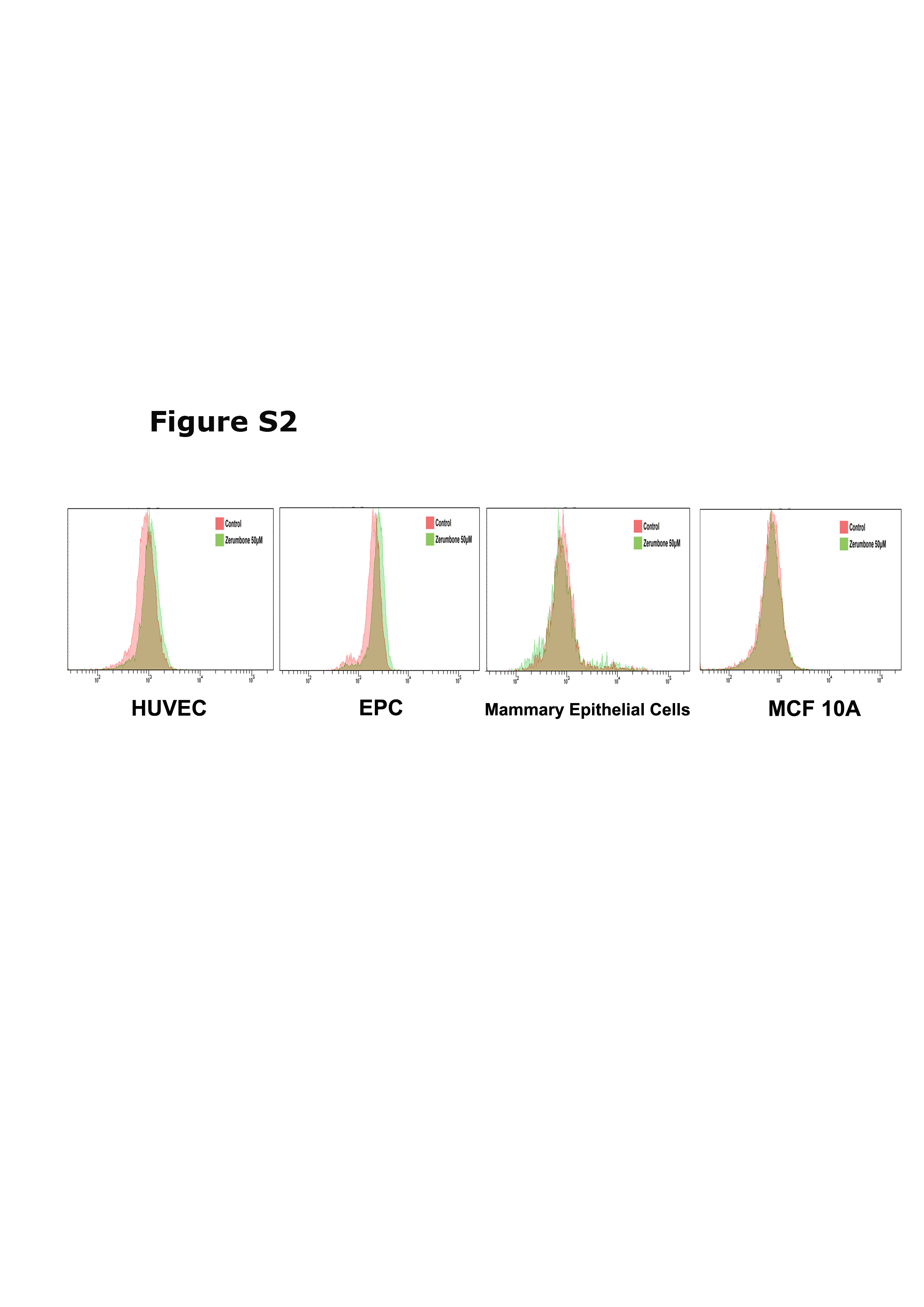

Supplement: Figure S2 — MCF-10 A, Human Mammary epithelial cells, Human Umbilical Cord Endothelial Cells and endothelial progenitor cells were treated with zerumbone 50 µM for 24 h. Then the cells were stained with t-BOC as described and analysed by flow cytometer. (TIF) [file pone.0059350.s002.tif]
