# Supplementary material for: Calpain and Reactive Oxygen Species Targets Bax for Mitochondrial Permeabilisation and Caspase Activation in Zerumbone Induced Apoptosis
Source: PLoS One. 2013 Apr 9;8(4):e59350. doi: 10.1371/journal.pone.0059350 (PMC3621898; doi:10.1371/journal.pone.0059350)
Supplement: Table S1 — List of antibodies and its respective dilutions. (DOCX) [file pone.0059350.s006.docx]

| **Antibody** | **Dilution** | **Cat.No.** | **Company** |
| --- | --- | --- | --- |
| Caspase 8 | 1:1000 | (#9746) | Cell Signaling Technologies |
| Bim | 1:1000 | (#2819) | Cell Signaling Technologies |
| PARP | 1:1000 | (#9542) | Cell Signaling Technologies |
| Akt | 1:1000 | (#9272) | Cell Signaling Technologies |
| Survivin | 1:1000 | (#2802) | Cell Signaling Technologies |
| XIAP | 1:1000 | (#2042) | Cell Signaling Technologies |
| Caspase 3 | 1:1000 | (#9662 ) | Cell Signaling Technologies |
| PUMA | 1:1000 | (#4976) | Cell Signaling Technologies |
| HSP90 | 1:500 | (SC-69703) | Santa Cruz Biotechnology,Inc |
| HSP 70 | 1:500 | (SC-59569) | Santa Cruz Biotechnology,Inc |
| HSC-70 | 1:500 | (SC-7298) | Santa Cruz Biotechnology,Inc |
| Cyclin D1 | 1:500 | (SC-56302) | Santa Cruz Biotechnology,Inc |
| Bax | 1:500 | (SC-493) | Santa Cruz Biotechnology,Inc |
| Bid | 1:500 | (SC-373939) | Santa Cruz Biotechnology,Inc |
| Beta Actin | 1:500 | (SC-130301) | Santa Cruz Biotechnology,Inc |

Table S1
