# Supplementary figures and images for: Correction: Calpain and Reactive Oxygen Species Targets Bax for Mitochondrial Permeabilisation and Caspase Activation in Zerumbone Induced Apoptosis
Source: PLoS One. 2022 Aug 23;17(8):e0273729. doi: 10.1371/journal.pone.0273729 (PMC9397940; doi:10.1371/journal.pone.0273729)

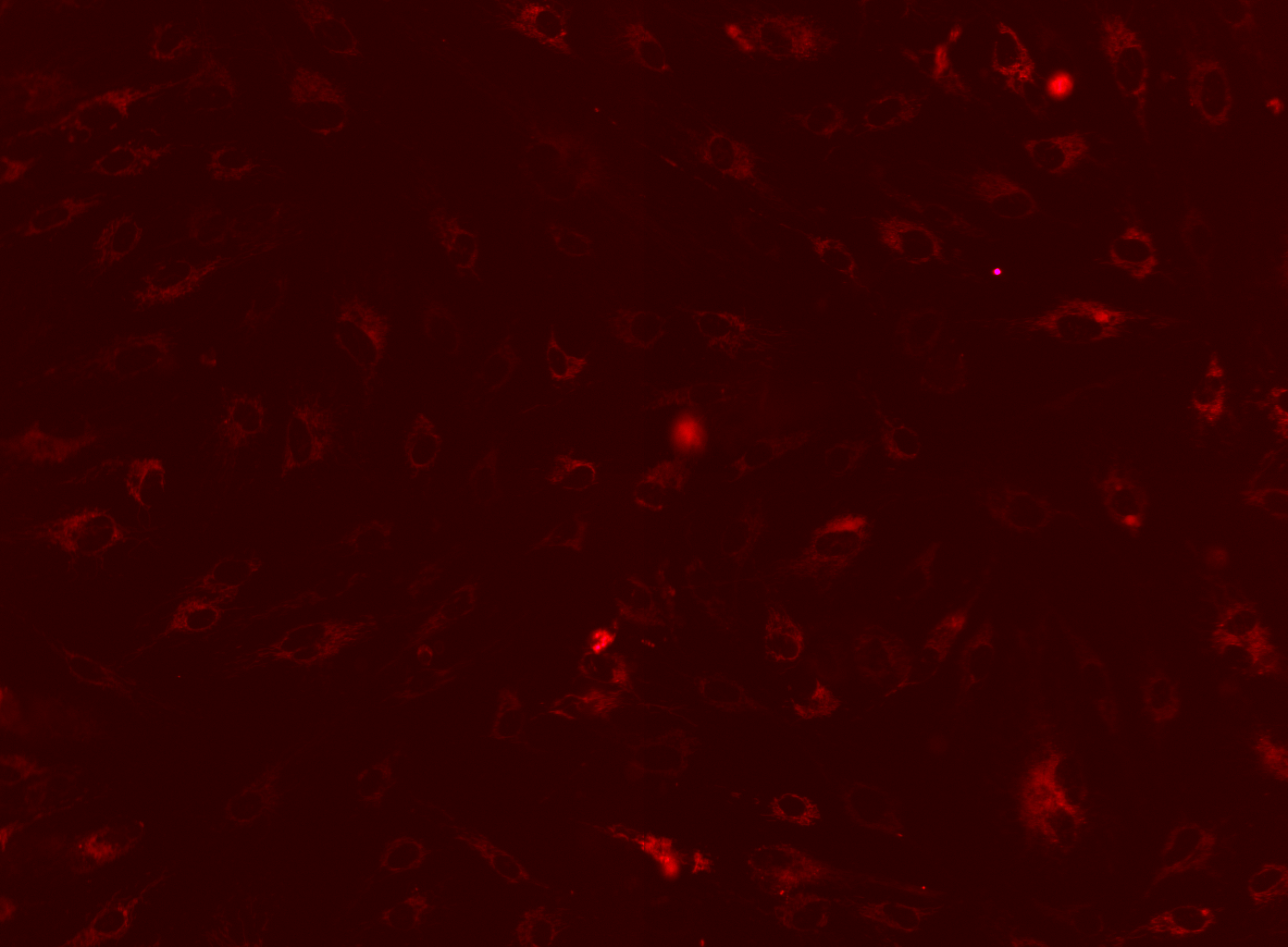

Supplement: S1 File — Images were captured using BD Pathway Bio-imager 435 and each file is a composite of four separate images captured as a 2x2 montage. (ZIP) [file pone.0273729.s001.zip › File S1. Raw image data for Figure 6H/EPC/12 hrs/Alexa 546 - n000000.bmp]

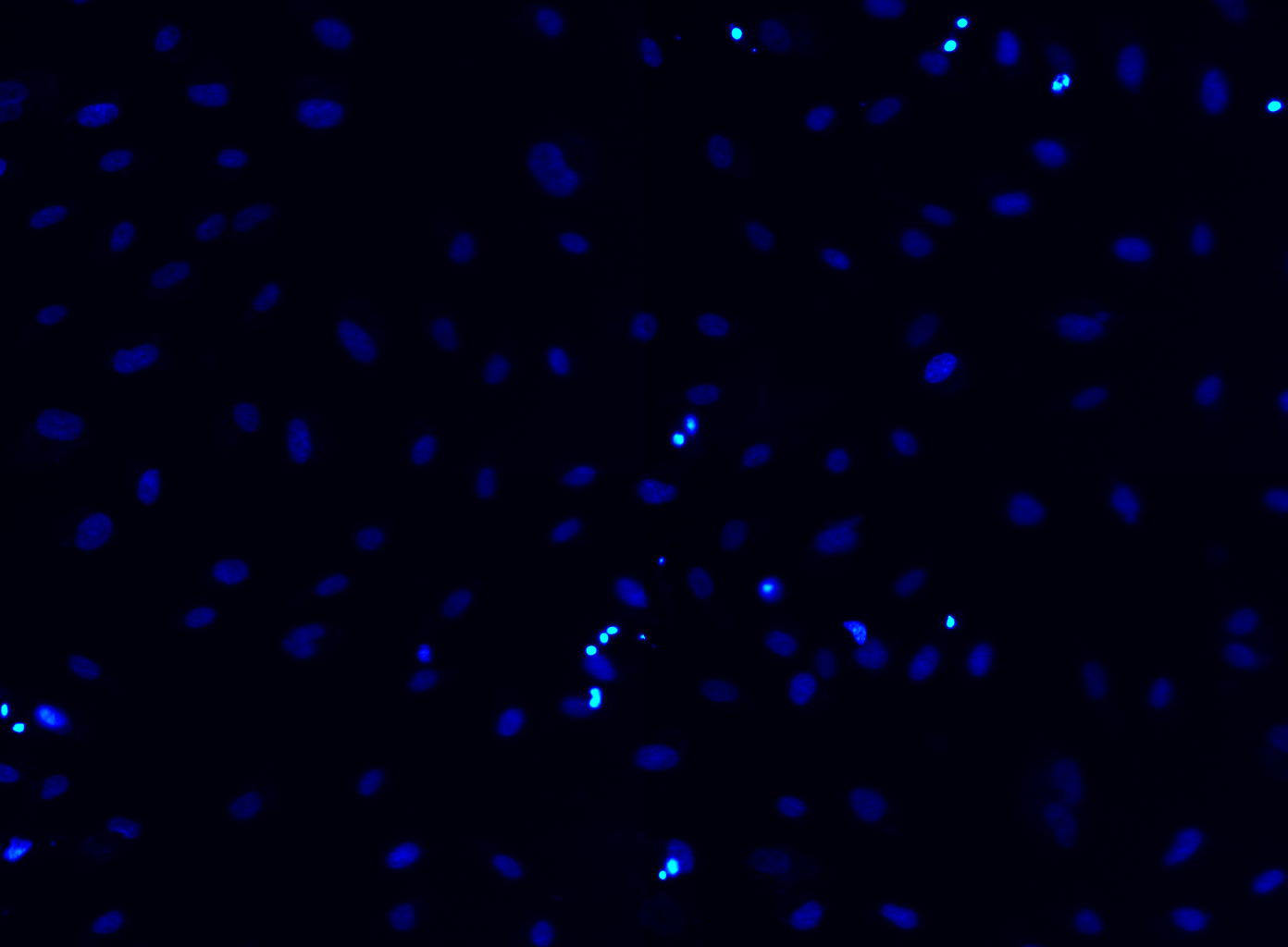

Supplement: S1 File — Images were captured using BD Pathway Bio-imager 435 and each file is a composite of four separate images captured as a 2x2 montage. (ZIP) [file pone.0273729.s001.zip › File S1. Raw image data for Figure 6H/EPC/12 hrs/Hoechst - n000000.bmp]

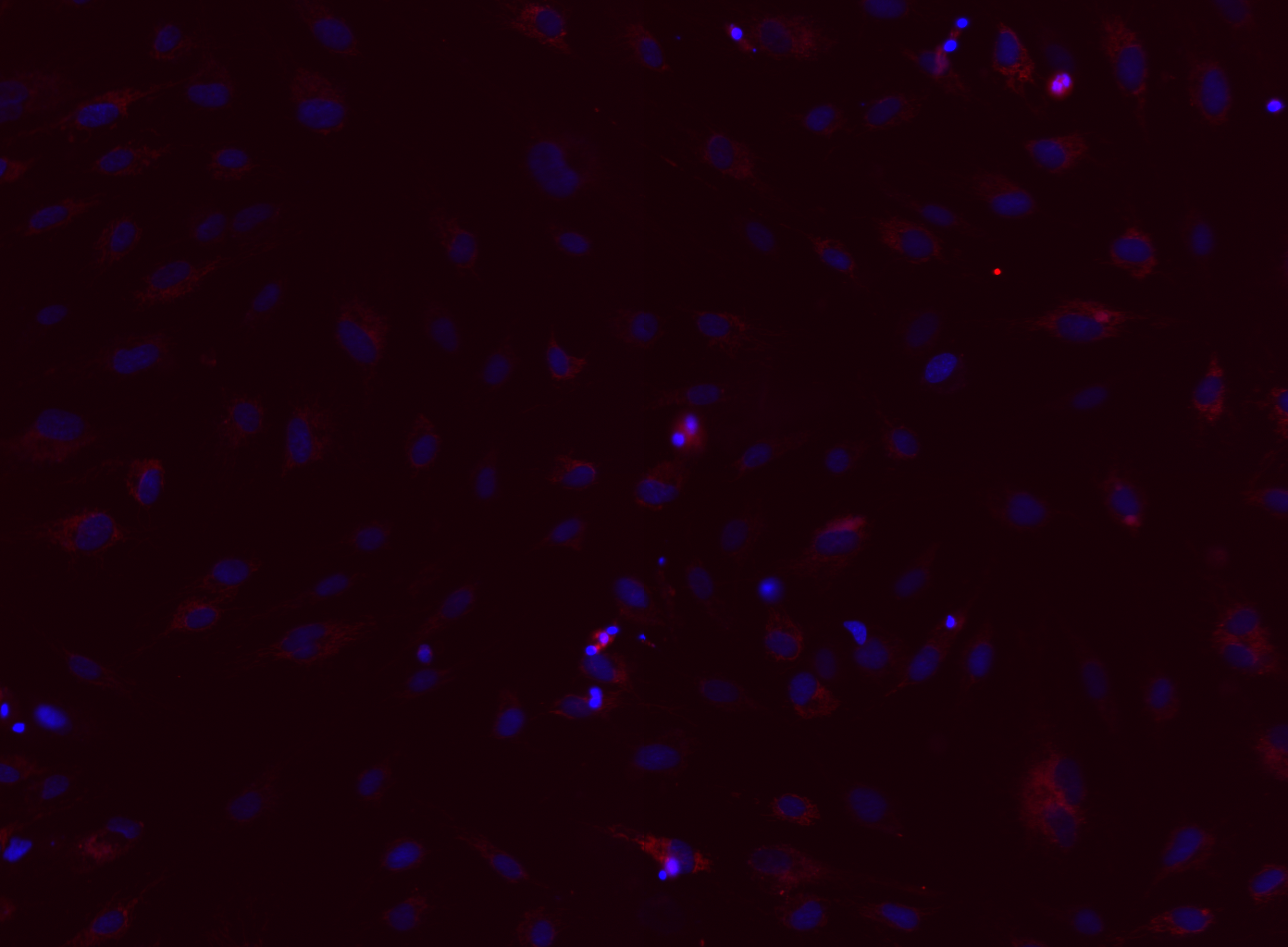

Supplement: S1 File — Images were captured using BD Pathway Bio-imager 435 and each file is a composite of four separate images captured as a 2x2 montage. (ZIP) [file pone.0273729.s001.zip › File S1. Raw image data for Figure 6H/EPC/12 hrs/merged.bmp]

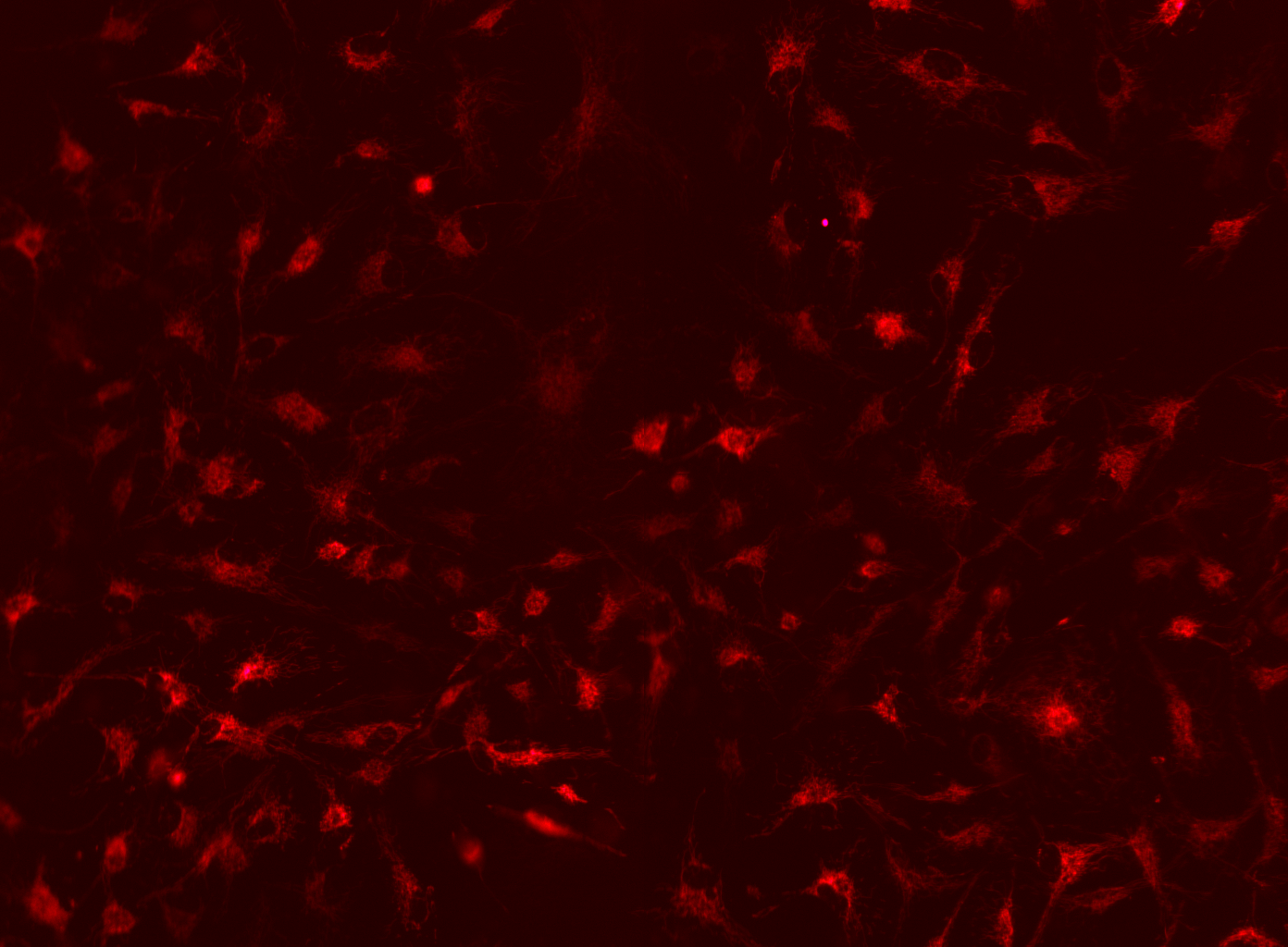

Supplement: S1 File — Images were captured using BD Pathway Bio-imager 435 and each file is a composite of four separate images captured as a 2x2 montage. (ZIP) [file pone.0273729.s001.zip › File S1. Raw image data for Figure 6H/EPC/24 h/Alexa 546 - n000000.bmp]

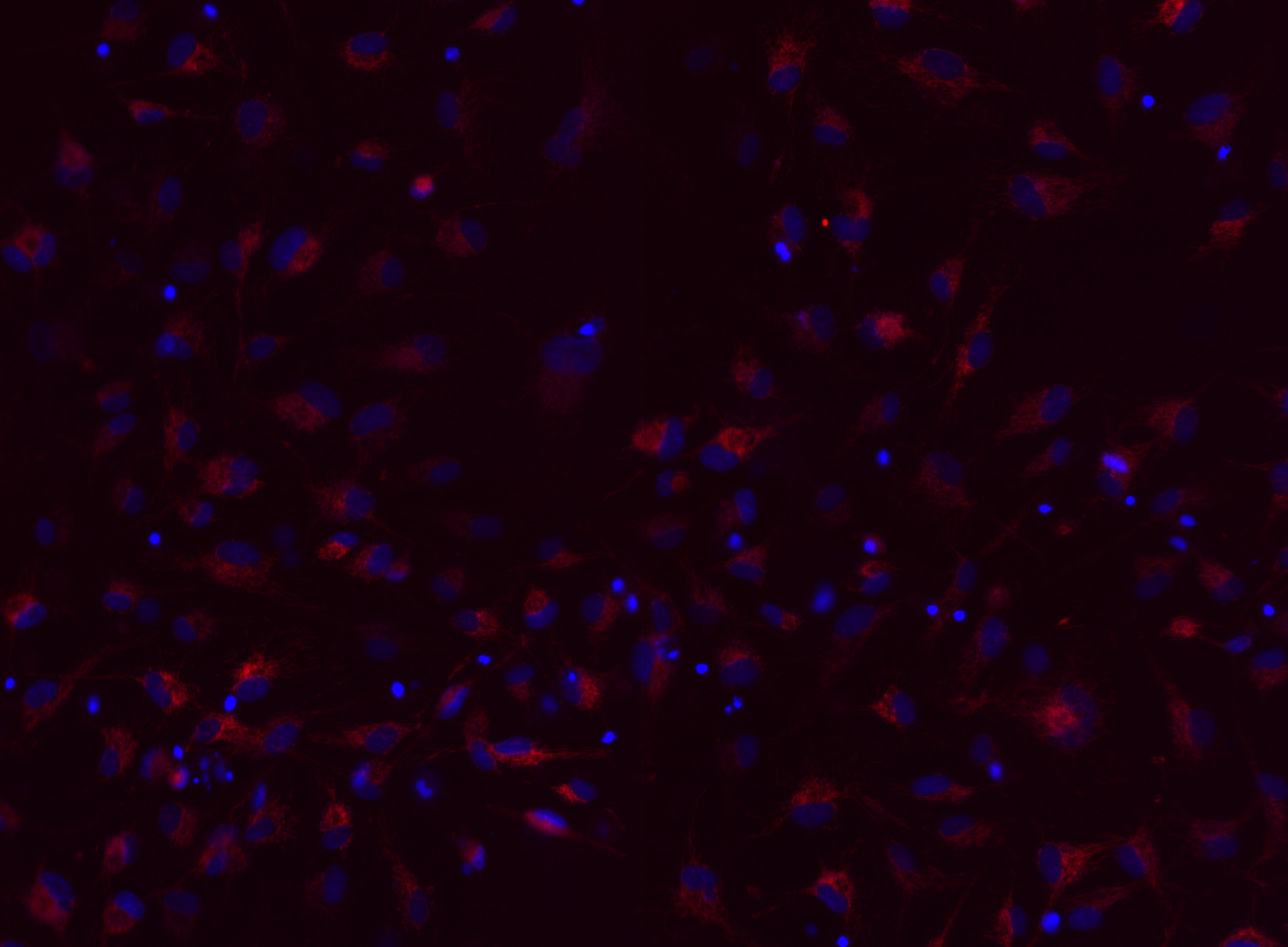

Supplement: S1 File — Images were captured using BD Pathway Bio-imager 435 and each file is a composite of four separate images captured as a 2x2 montage. (ZIP) [file pone.0273729.s001.zip › File S1. Raw image data for Figure 6H/EPC/24 h/h7.bmp]

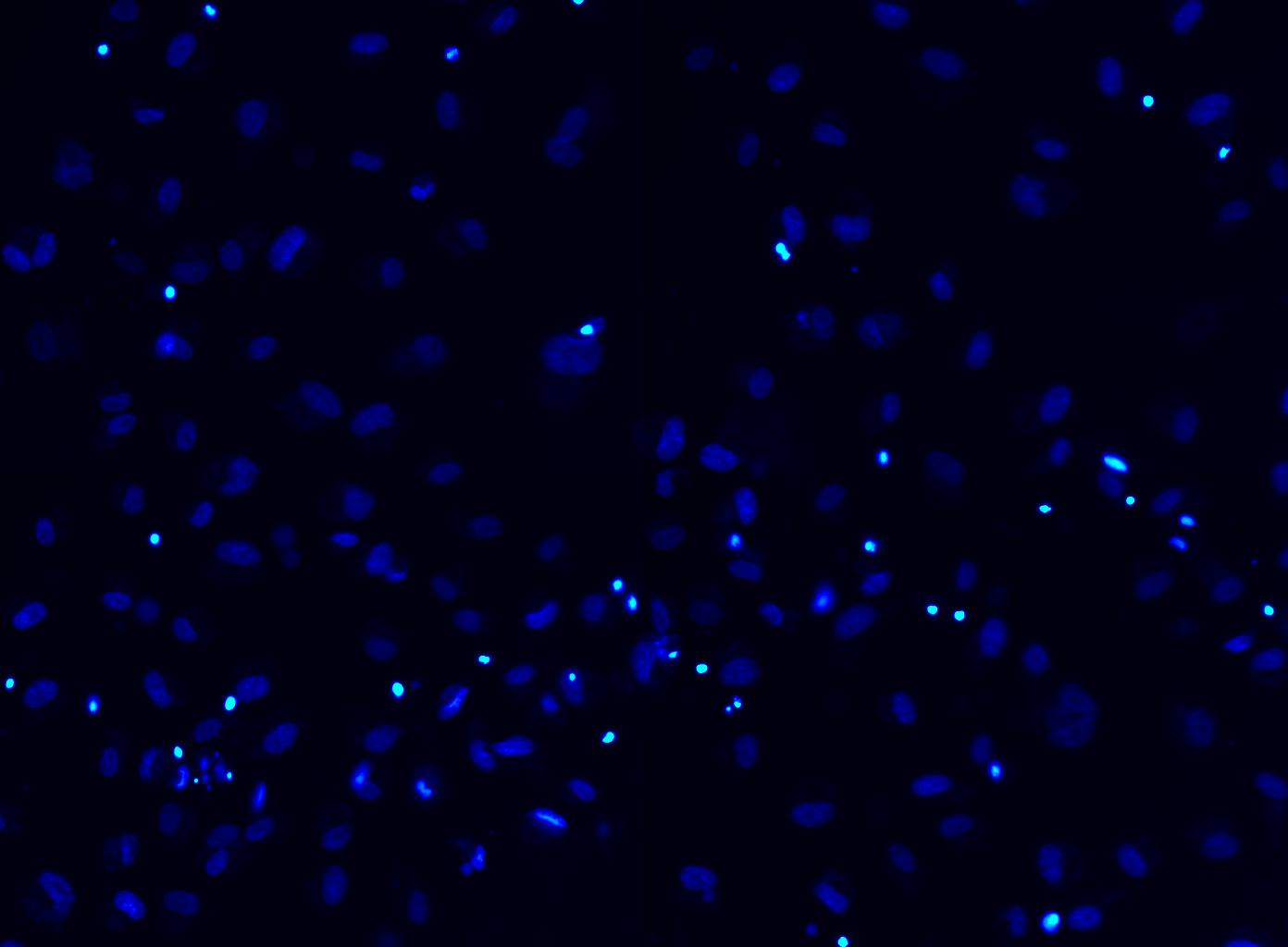

Supplement: S1 File — Images were captured using BD Pathway Bio-imager 435 and each file is a composite of four separate images captured as a 2x2 montage. (ZIP) [file pone.0273729.s001.zip › File S1. Raw image data for Figure 6H/EPC/24 h/Hoechst - n000000.bmp]

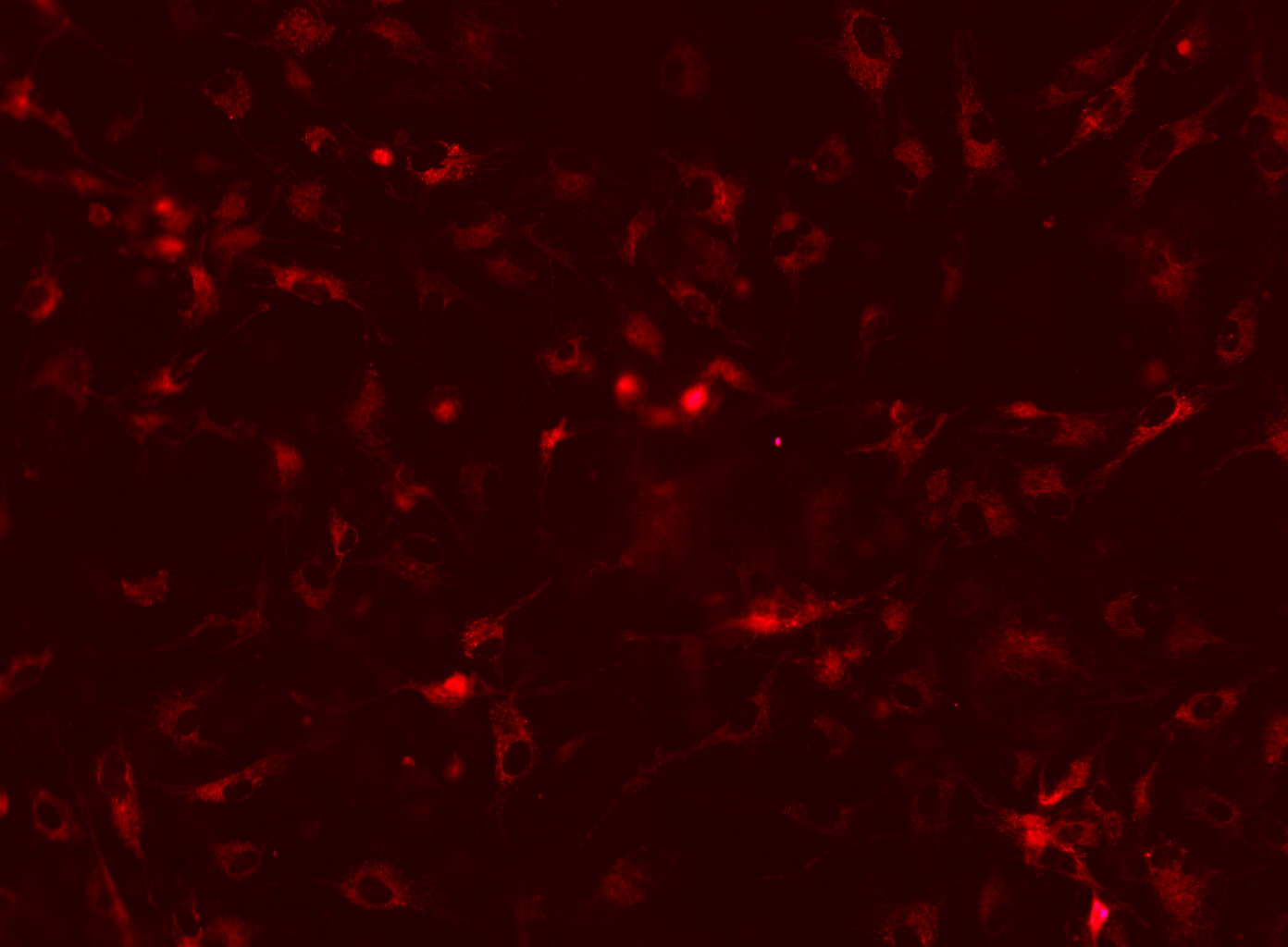

Supplement: S1 File — Images were captured using BD Pathway Bio-imager 435 and each file is a composite of four separate images captured as a 2x2 montage. (ZIP) [file pone.0273729.s001.zip › File S1. Raw image data for Figure 6H/EPC/48 h/Alexa 546 - n000000.bmp]

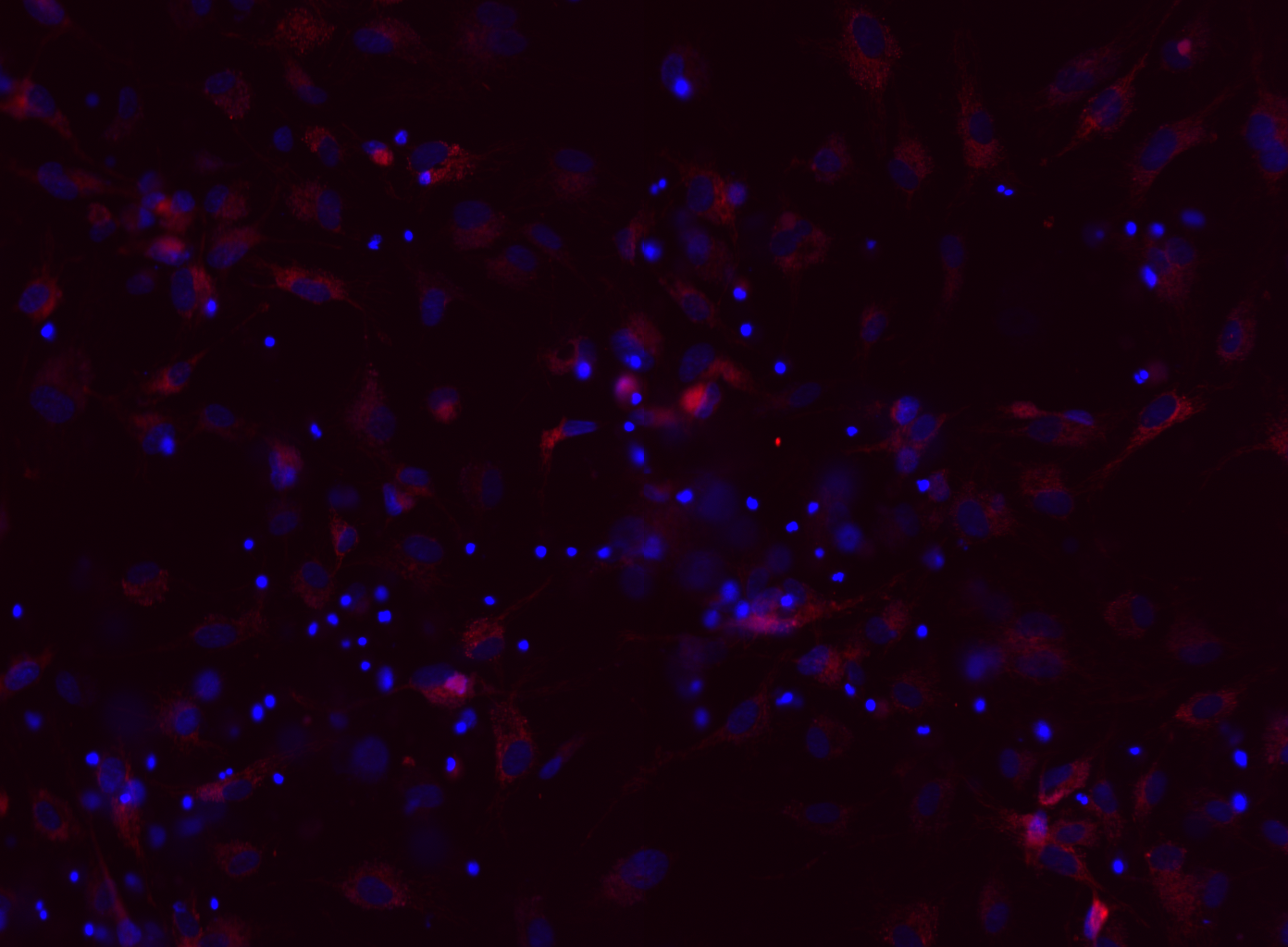

Supplement: S1 File — Images were captured using BD Pathway Bio-imager 435 and each file is a composite of four separate images captured as a 2x2 montage. (ZIP) [file pone.0273729.s001.zip › File S1. Raw image data for Figure 6H/EPC/48 h/h7.bmp]

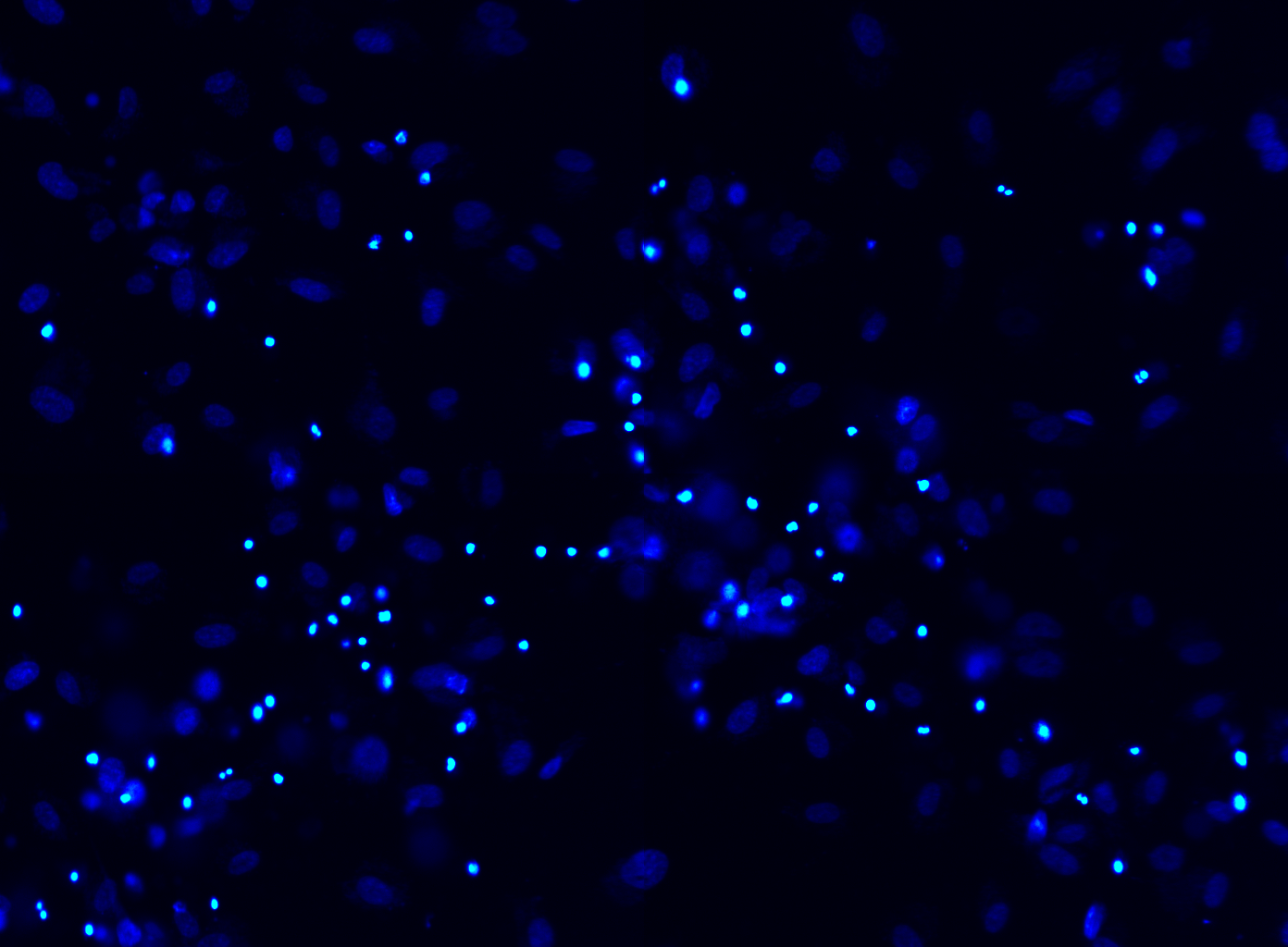

Supplement: S1 File — Images were captured using BD Pathway Bio-imager 435 and each file is a composite of four separate images captured as a 2x2 montage. (ZIP) [file pone.0273729.s001.zip › File S1. Raw image data for Figure 6H/EPC/48 h/Hoechst - n000000.bmp]

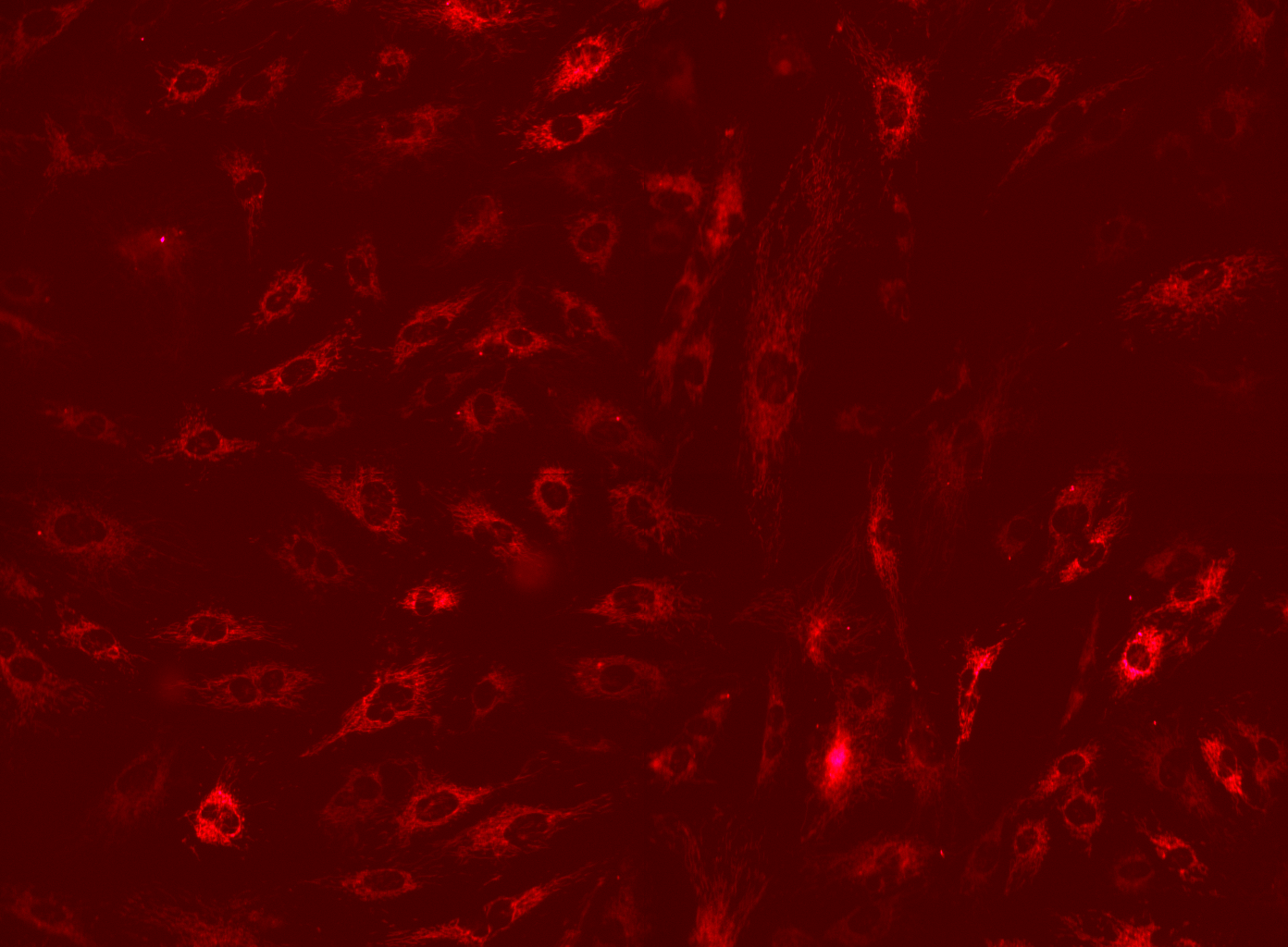

Supplement: S1 File — Images were captured using BD Pathway Bio-imager 435 and each file is a composite of four separate images captured as a 2x2 montage. (ZIP) [file pone.0273729.s001.zip › File S1. Raw image data for Figure 6H/EPC/CONTROL/Alexa 546 - n000000.bmp]

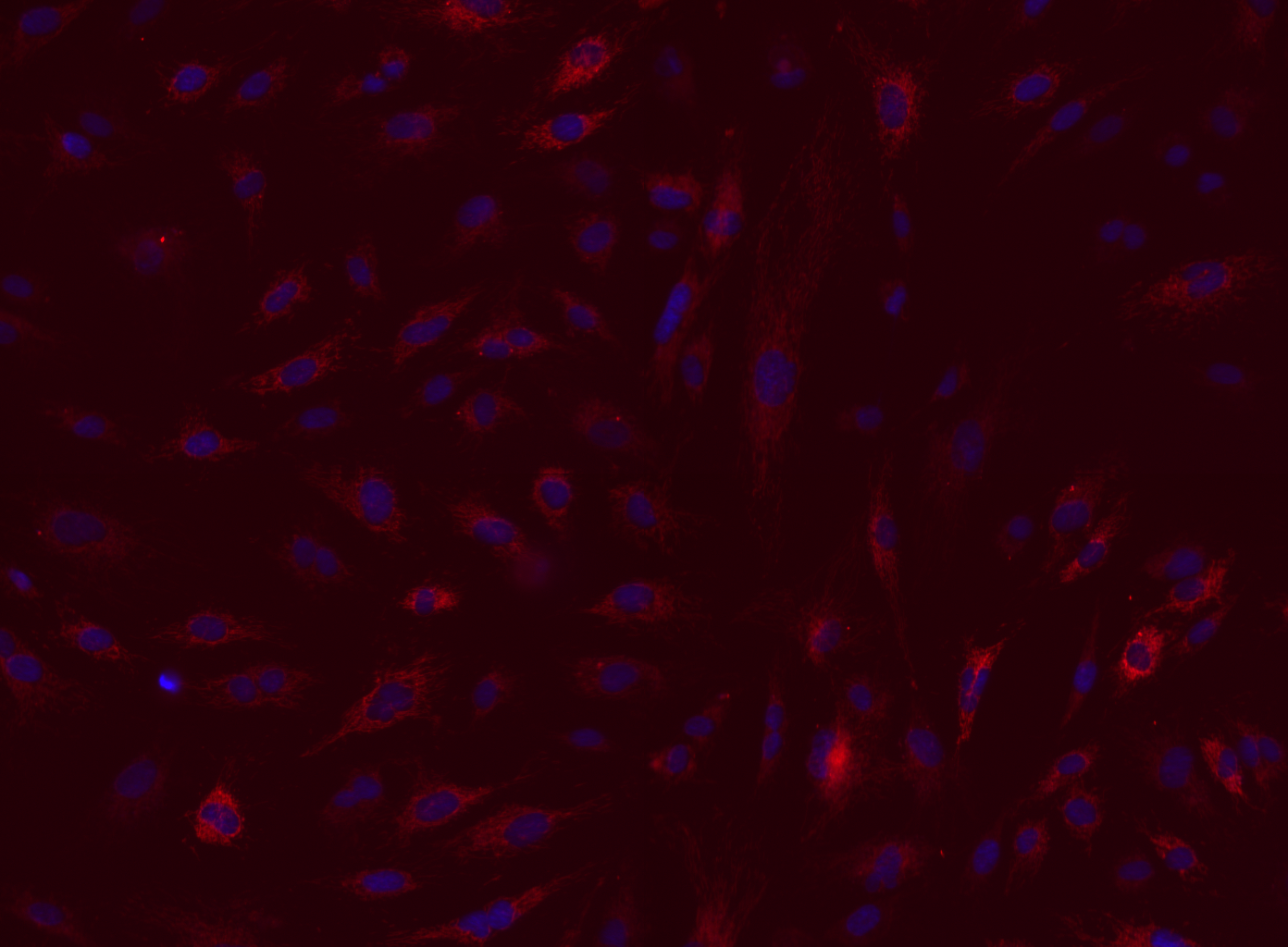

Supplement: S1 File — Images were captured using BD Pathway Bio-imager 435 and each file is a composite of four separate images captured as a 2x2 montage. (ZIP) [file pone.0273729.s001.zip › File S1. Raw image data for Figure 6H/EPC/CONTROL/h7.bmp]

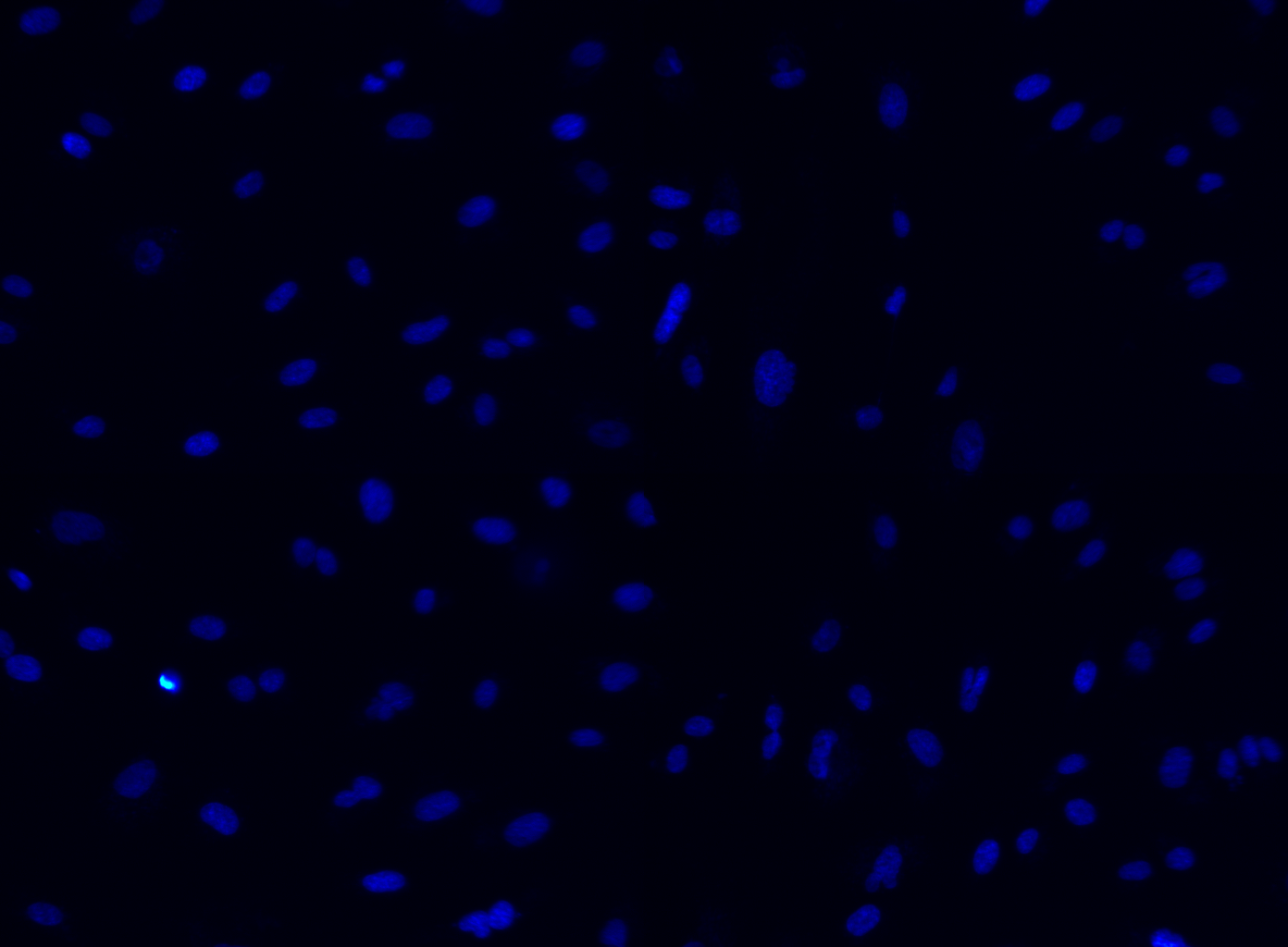

Supplement: S1 File — Images were captured using BD Pathway Bio-imager 435 and each file is a composite of four separate images captured as a 2x2 montage. (ZIP) [file pone.0273729.s001.zip › File S1. Raw image data for Figure 6H/EPC/CONTROL/Hoechst - n000000.bmp]

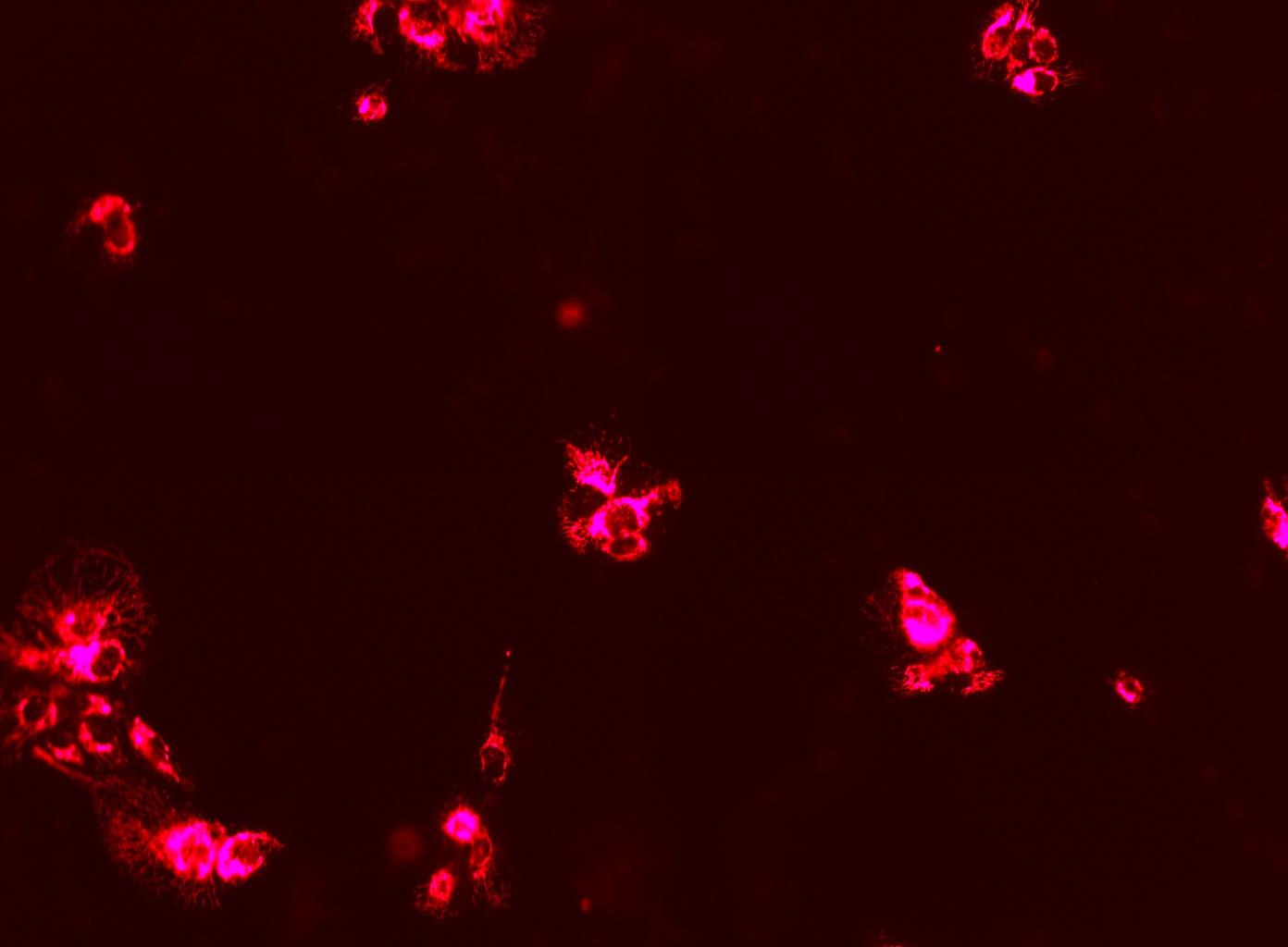

Supplement: S1 File — Images were captured using BD Pathway Bio-imager 435 and each file is a composite of four separate images captured as a 2x2 montage. (ZIP) [file pone.0273729.s001.zip › File S1. Raw image data for Figure 6H/MCF 10A/12 h/Alexa 546 - n000000.bmp]

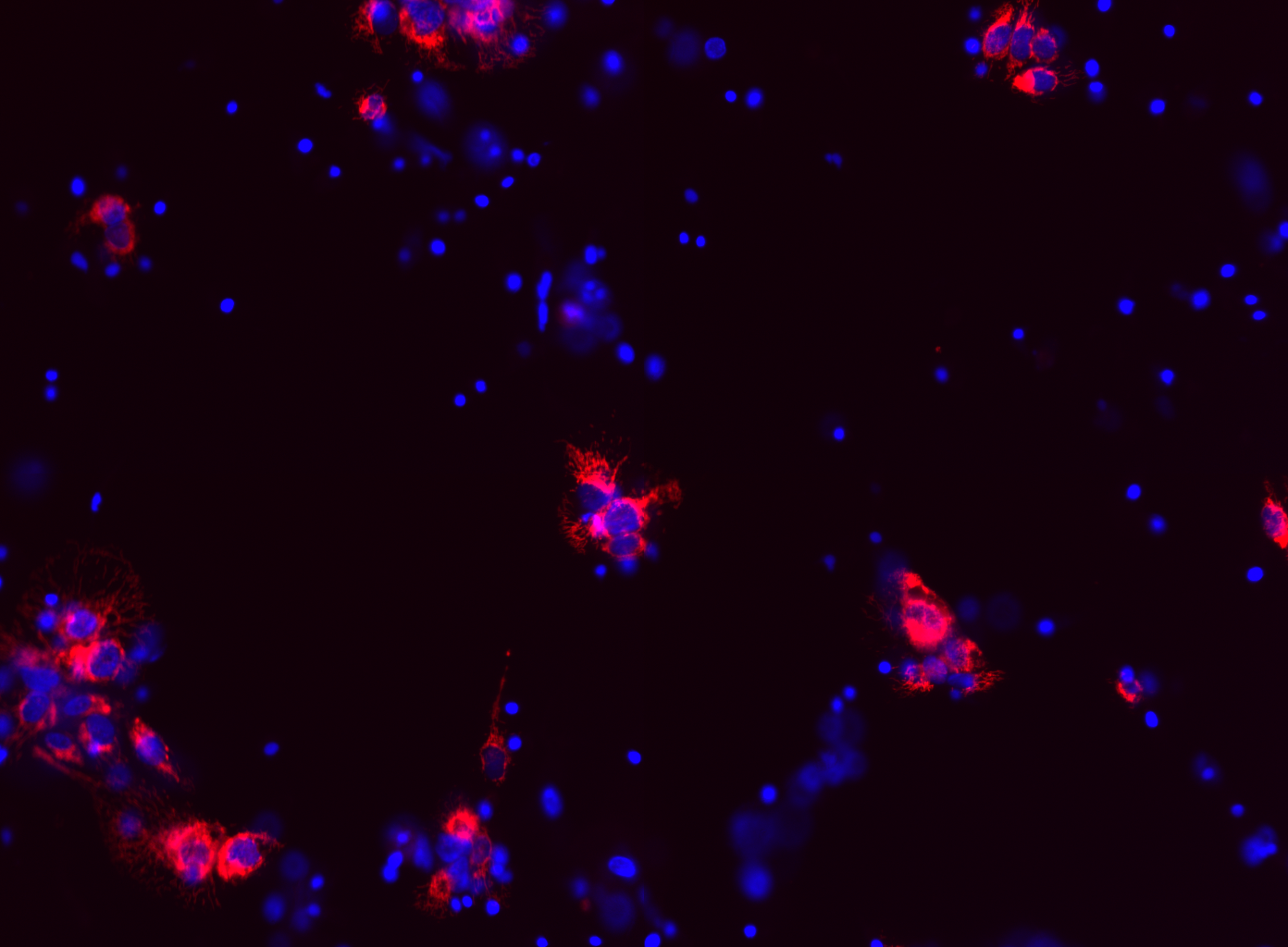

Supplement: S1 File — Images were captured using BD Pathway Bio-imager 435 and each file is a composite of four separate images captured as a 2x2 montage. (ZIP) [file pone.0273729.s001.zip › File S1. Raw image data for Figure 6H/MCF 10A/12 h/h7.bmp]

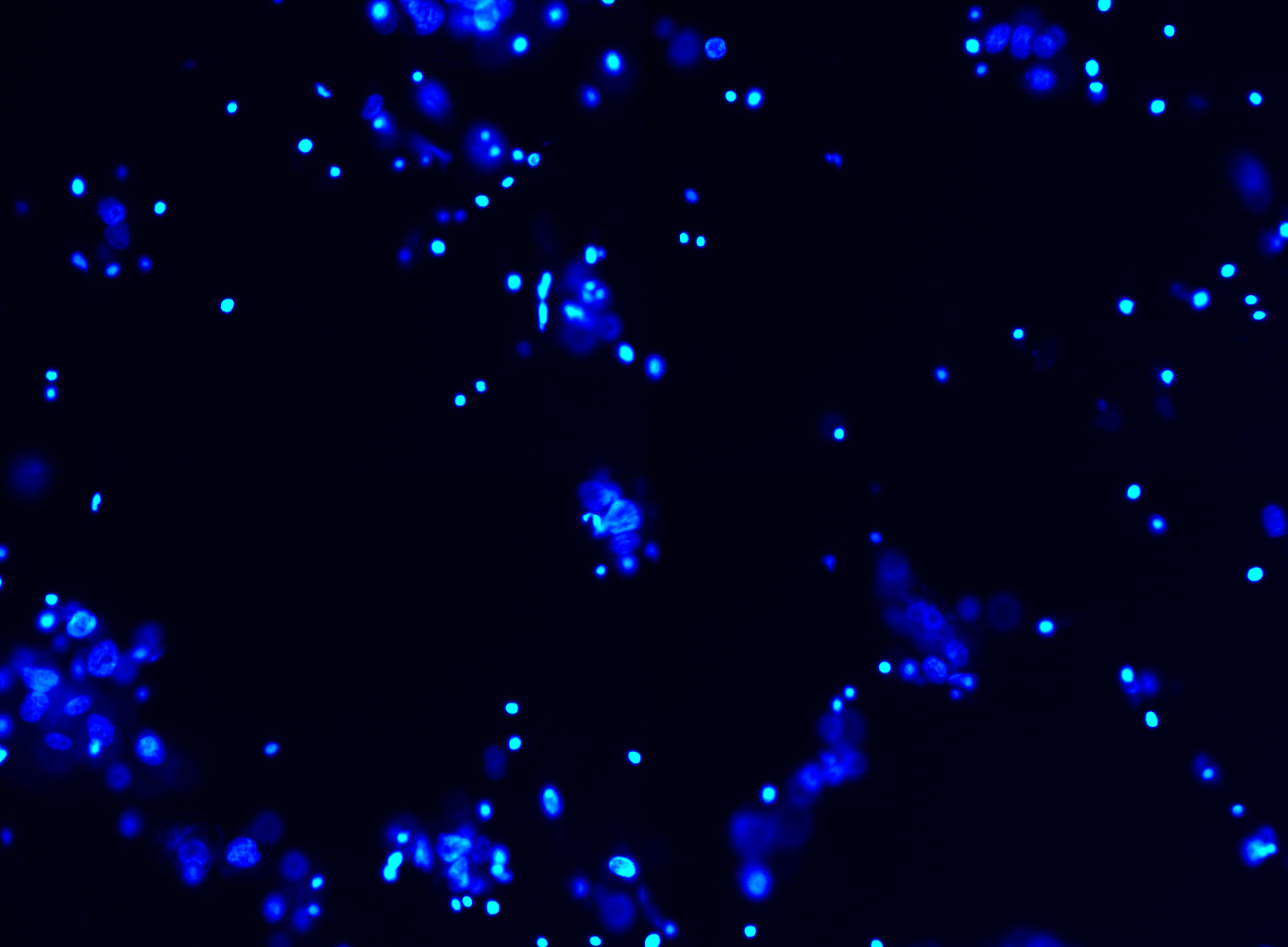

Supplement: S1 File — Images were captured using BD Pathway Bio-imager 435 and each file is a composite of four separate images captured as a 2x2 montage. (ZIP) [file pone.0273729.s001.zip › File S1. Raw image data for Figure 6H/MCF 10A/12 h/Hoechst - n000000.bmp]

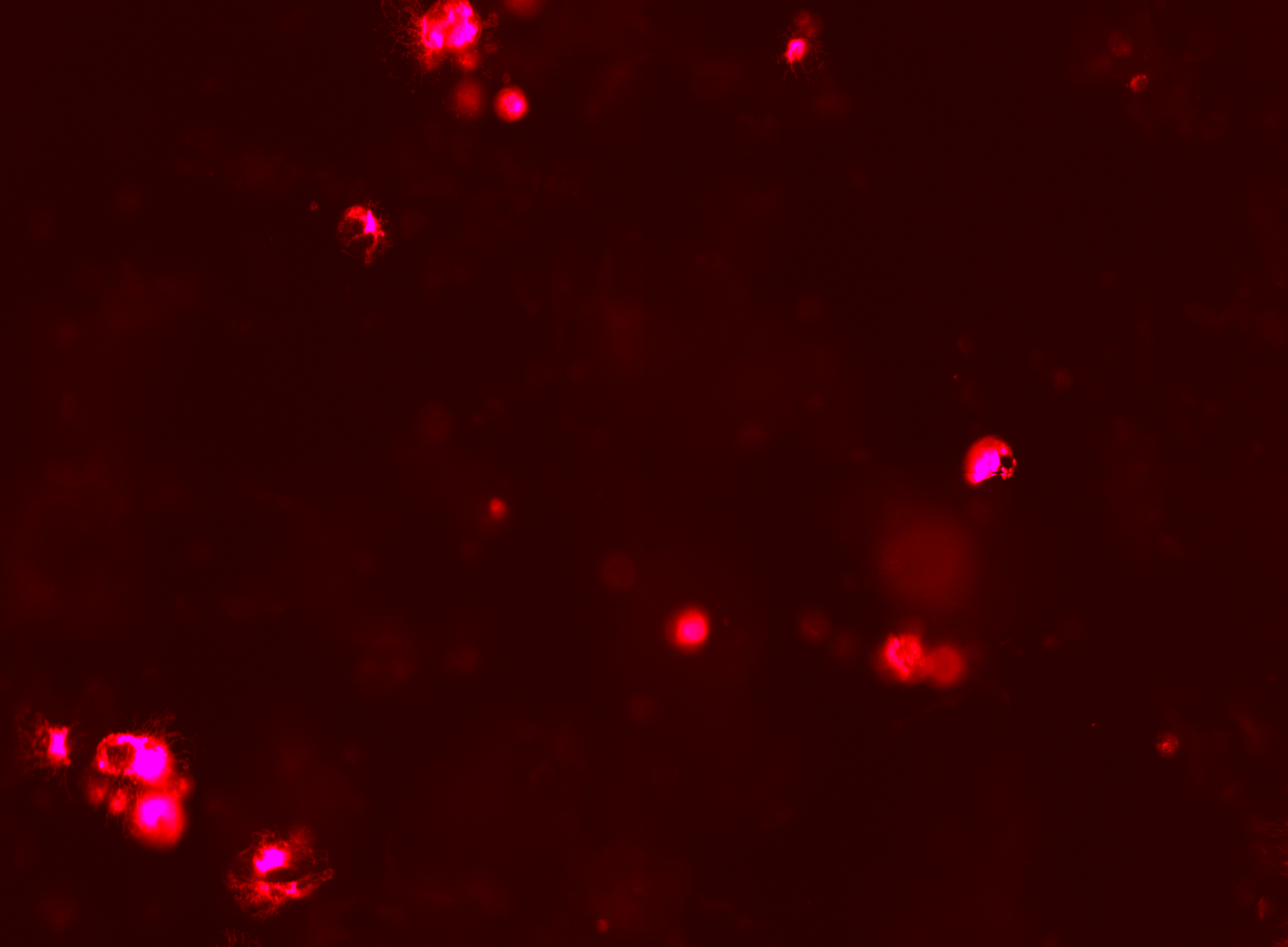

Supplement: S1 File — Images were captured using BD Pathway Bio-imager 435 and each file is a composite of four separate images captured as a 2x2 montage. (ZIP) [file pone.0273729.s001.zip › File S1. Raw image data for Figure 6H/MCF 10A/24 h/Alexa 546 - n000000.bmp]

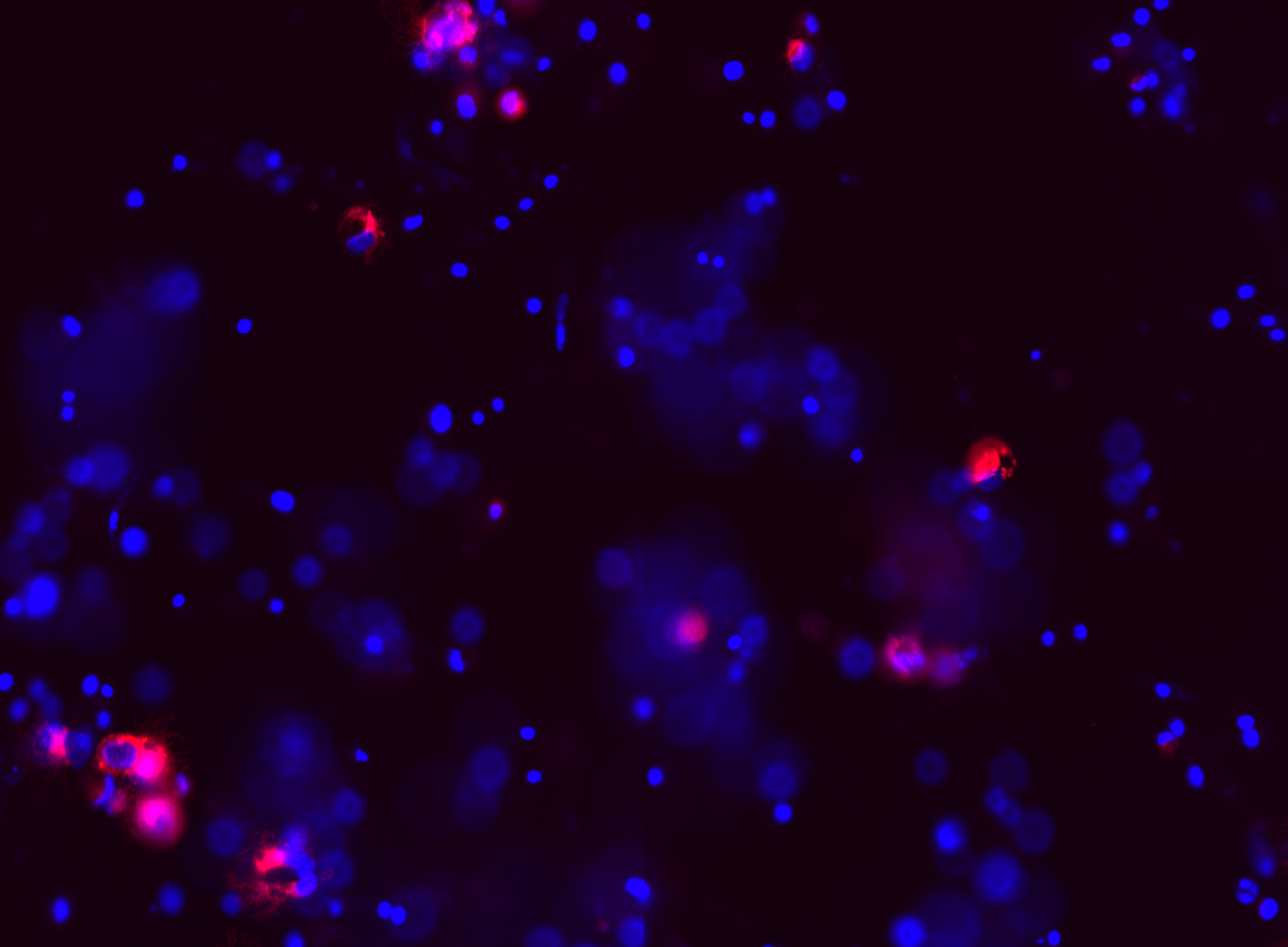

Supplement: S1 File — Images were captured using BD Pathway Bio-imager 435 and each file is a composite of four separate images captured as a 2x2 montage. (ZIP) [file pone.0273729.s001.zip › File S1. Raw image data for Figure 6H/MCF 10A/24 h/h7.bmp]

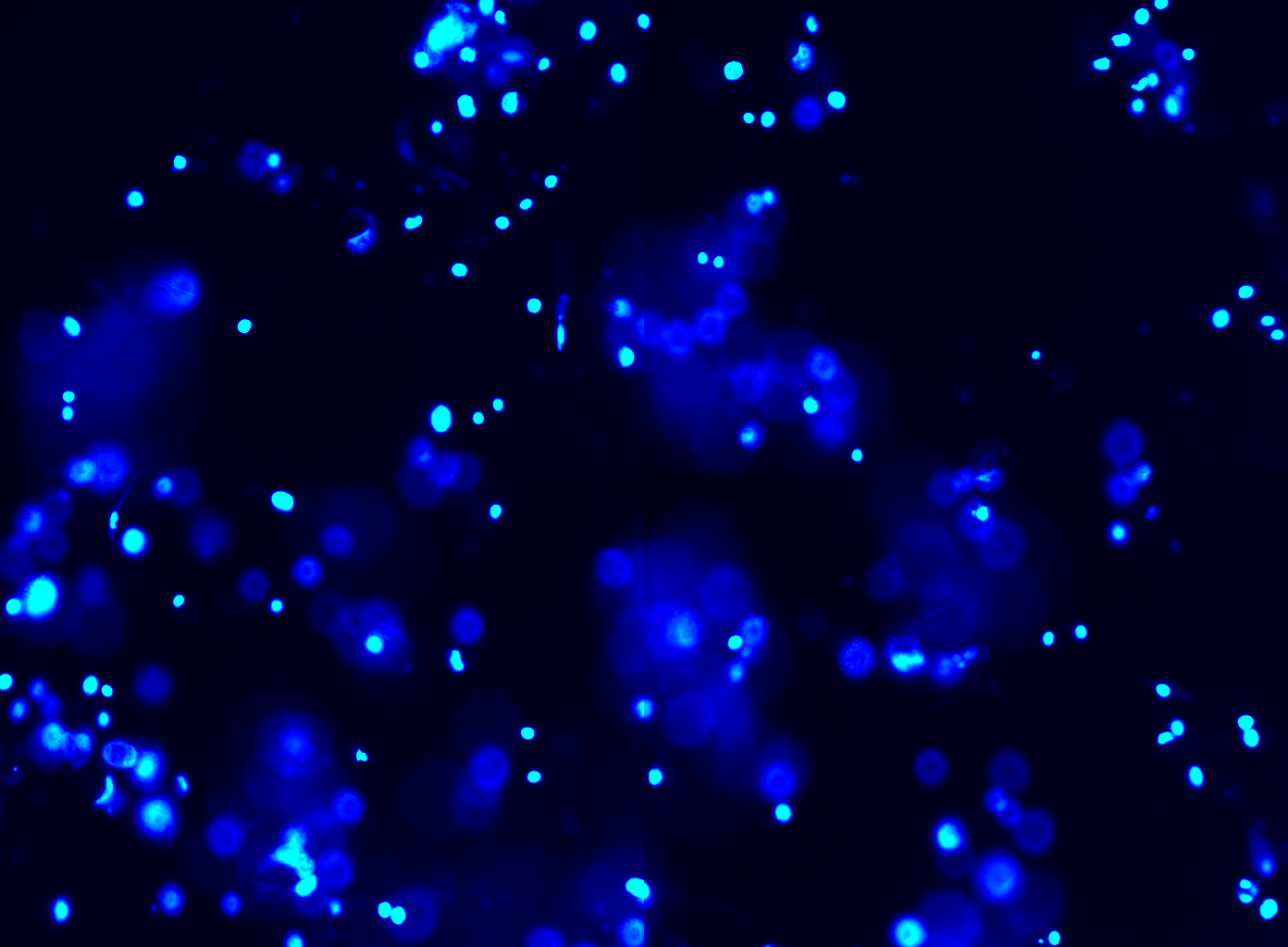

Supplement: S1 File — Images were captured using BD Pathway Bio-imager 435 and each file is a composite of four separate images captured as a 2x2 montage. (ZIP) [file pone.0273729.s001.zip › File S1. Raw image data for Figure 6H/MCF 10A/24 h/Hoechst -.bmp]

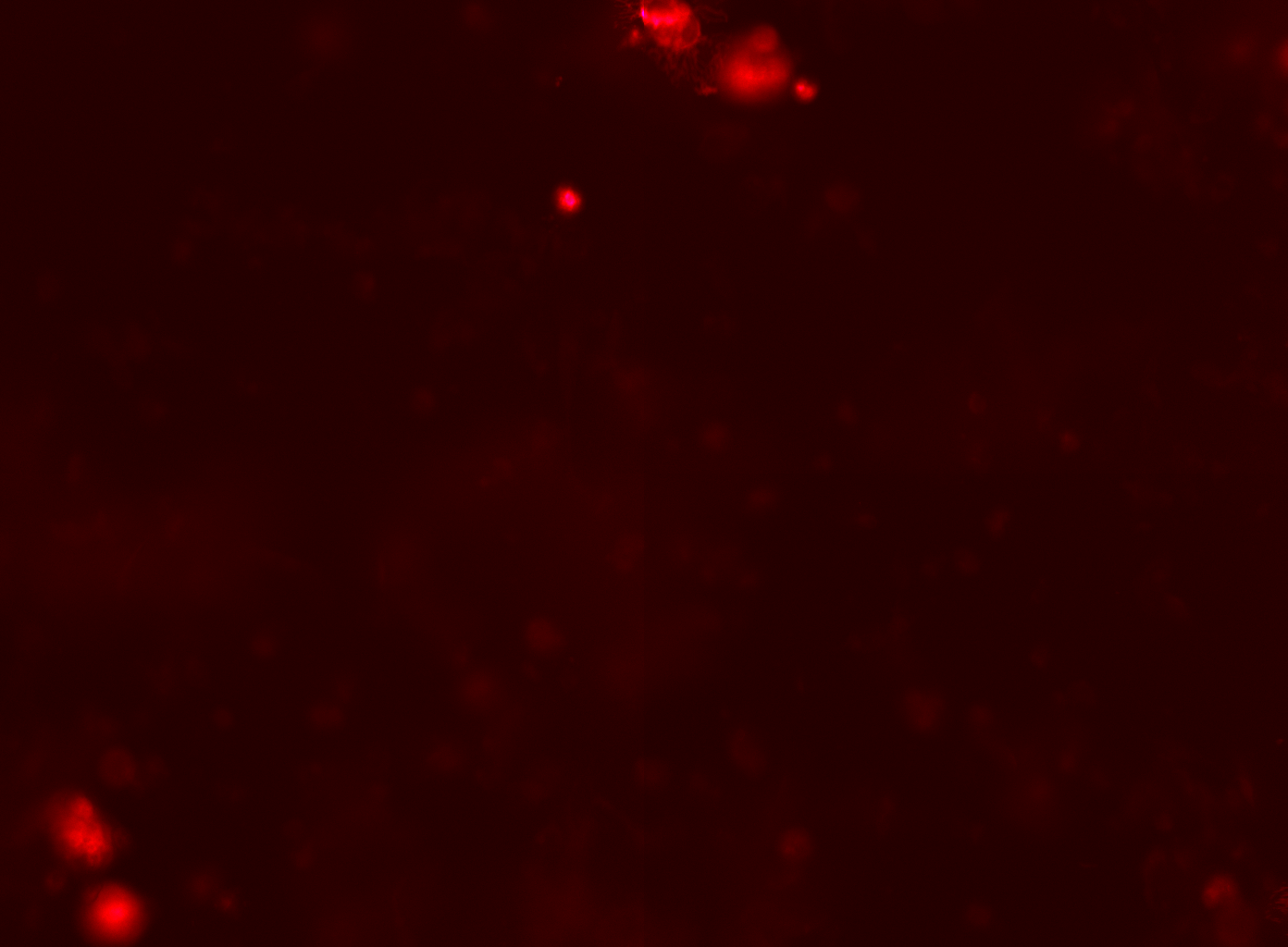

Supplement: S1 File — Images were captured using BD Pathway Bio-imager 435 and each file is a composite of four separate images captured as a 2x2 montage. (ZIP) [file pone.0273729.s001.zip › File S1. Raw image data for Figure 6H/MCF 10A/48 h/Alexa 546 - n000000.bmp]

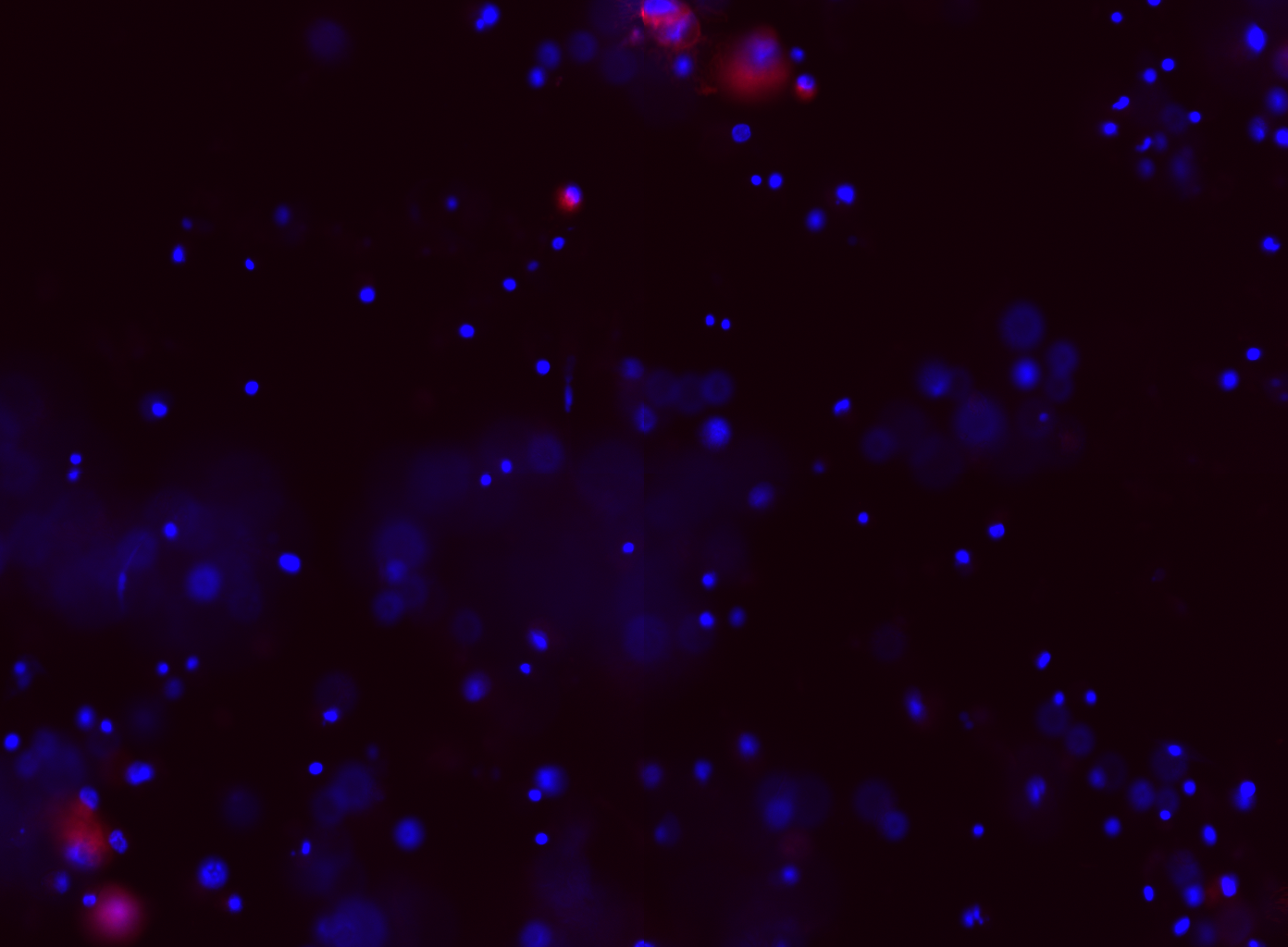

Supplement: S1 File — Images were captured using BD Pathway Bio-imager 435 and each file is a composite of four separate images captured as a 2x2 montage. (ZIP) [file pone.0273729.s001.zip › File S1. Raw image data for Figure 6H/MCF 10A/48 h/h7.bmp]

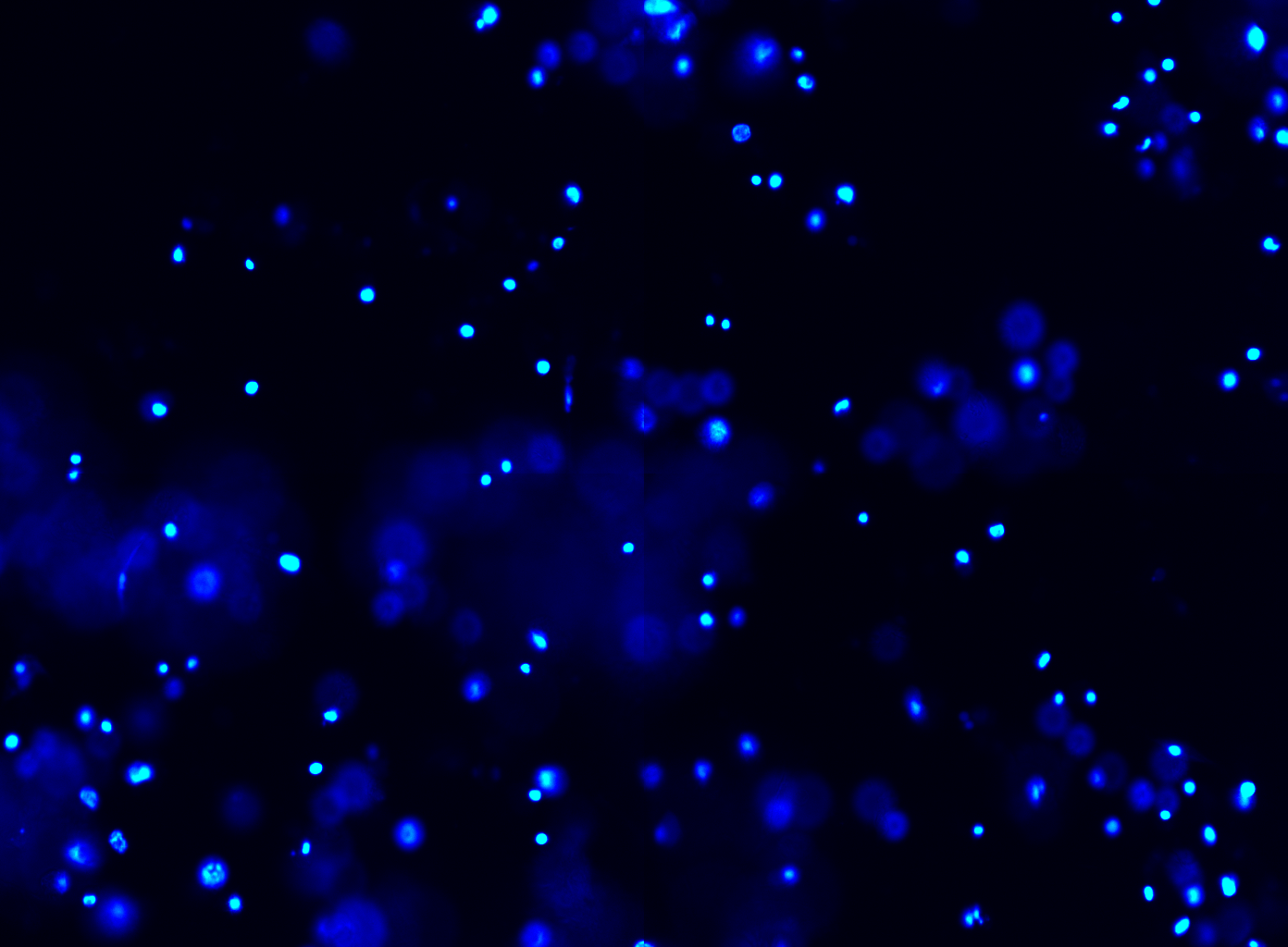

Supplement: S1 File — Images were captured using BD Pathway Bio-imager 435 and each file is a composite of four separate images captured as a 2x2 montage. (ZIP) [file pone.0273729.s001.zip › File S1. Raw image data for Figure 6H/MCF 10A/48 h/Hoechst - n000000.bmp]

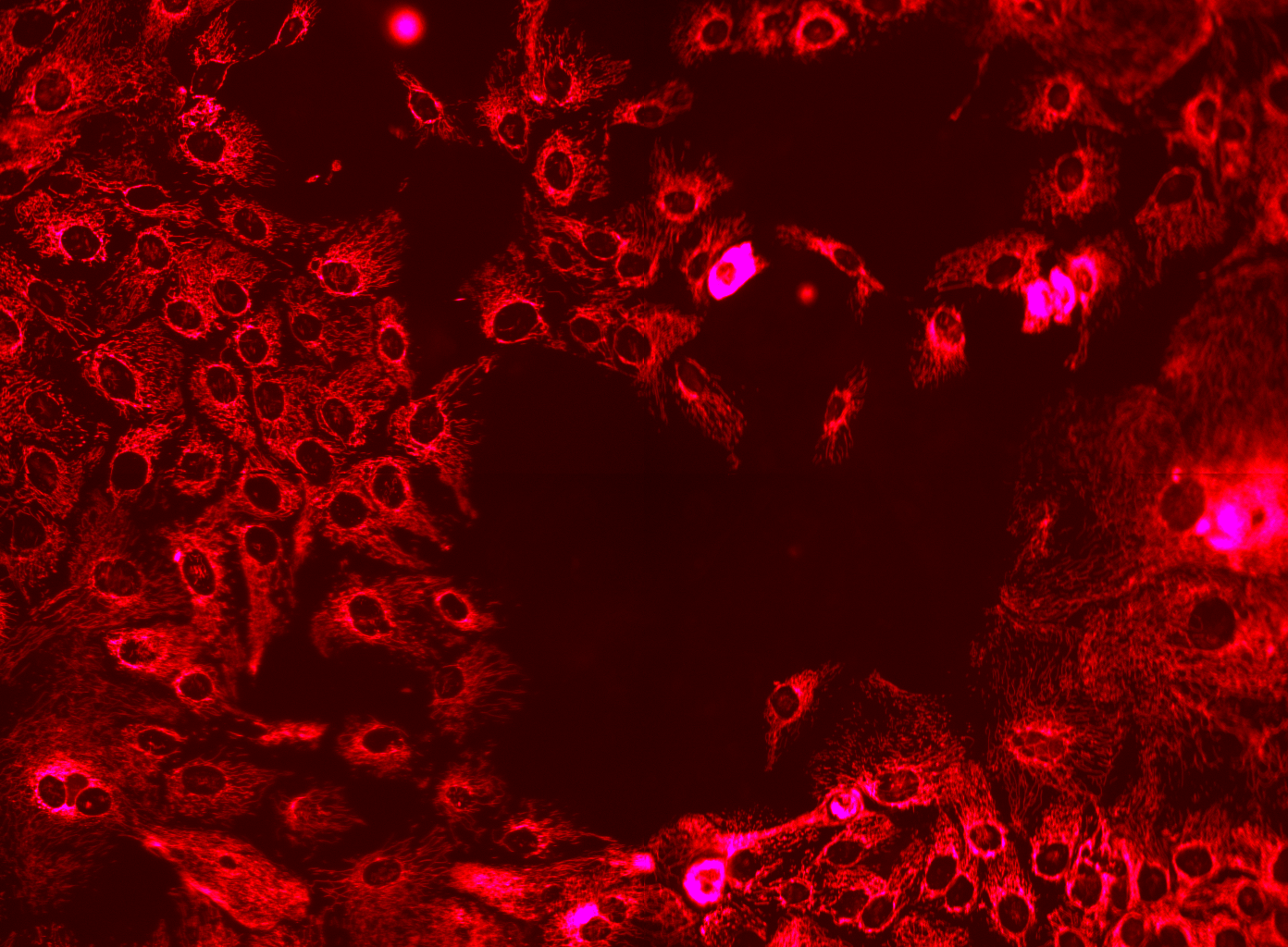

Supplement: S1 File — Images were captured using BD Pathway Bio-imager 435 and each file is a composite of four separate images captured as a 2x2 montage. (ZIP) [file pone.0273729.s001.zip › File S1. Raw image data for Figure 6H/MCF 10A/CONTROL/Alexa 546 - n000000.bmp]

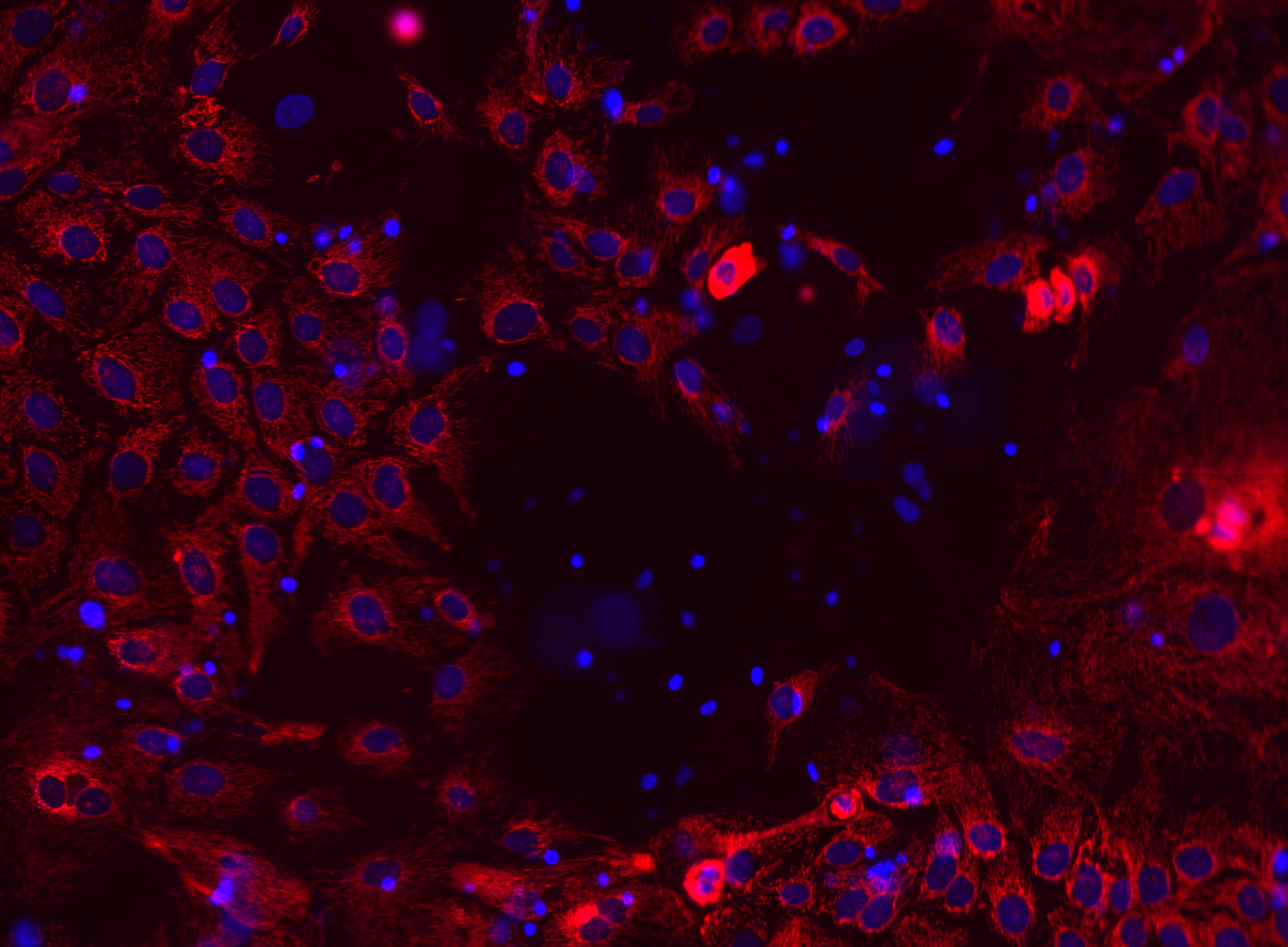

Supplement: S1 File — Images were captured using BD Pathway Bio-imager 435 and each file is a composite of four separate images captured as a 2x2 montage. (ZIP) [file pone.0273729.s001.zip › File S1. Raw image data for Figure 6H/MCF 10A/CONTROL/h7.bmp]

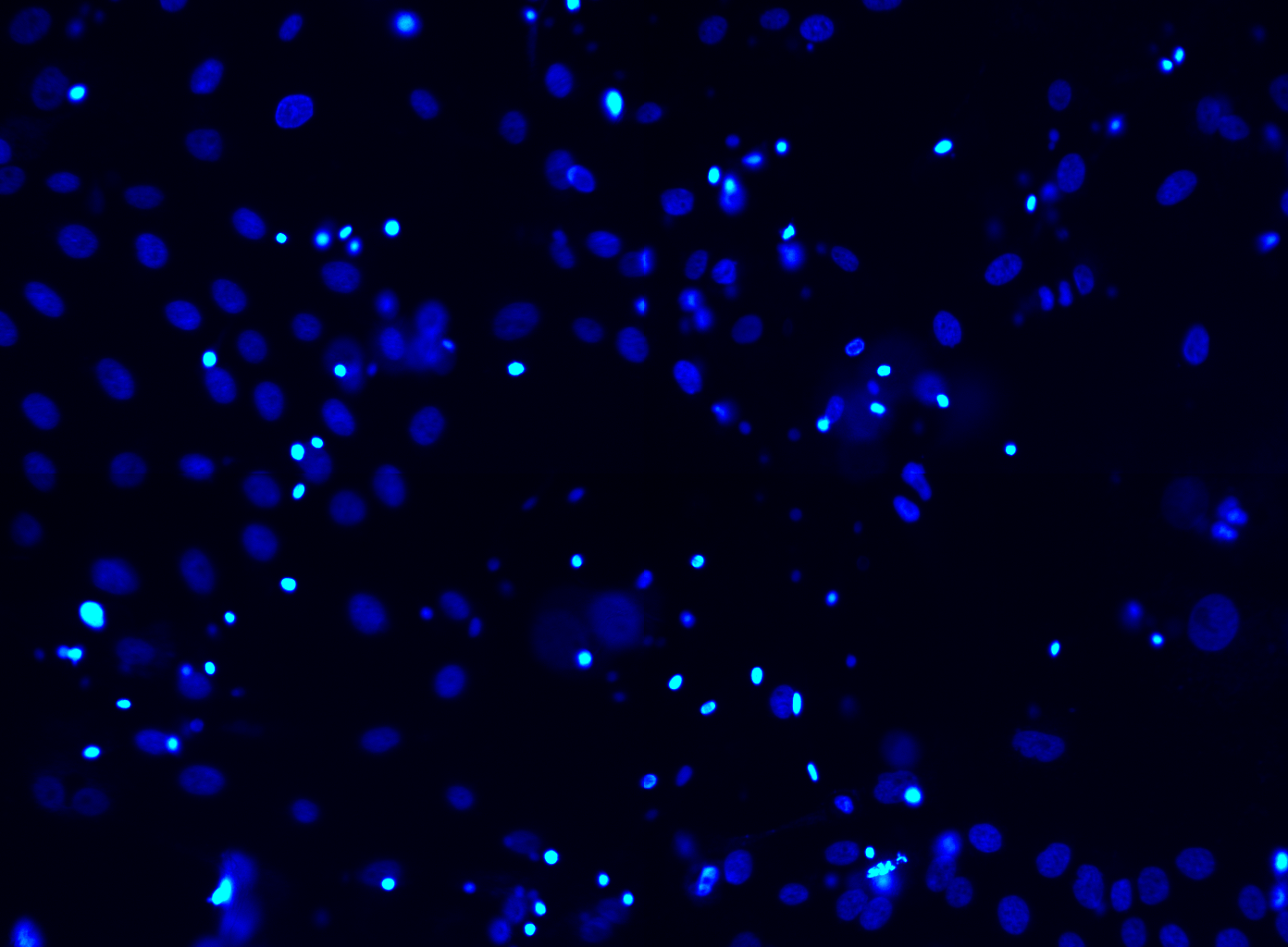

Supplement: S1 File — Images were captured using BD Pathway Bio-imager 435 and each file is a composite of four separate images captured as a 2x2 montage. (ZIP) [file pone.0273729.s001.zip › File S1. Raw image data for Figure 6H/MCF 10A/CONTROL/Hoechst - n000000.bmp]
