# Supplementary material for: Correction: Calpain and Reactive Oxygen Species Targets Bax for Mitochondrial Permeabilisation and Caspase Activation in Zerumbone Induced Apoptosis
Source: PLoS One. 2022 Aug 23;17(8):e0273729. doi: 10.1371/journal.pone.0273729 (PMC9397940; doi:10.1371/journal.pone.0273729)
Supplement: S2 File — (DOCX) [file pone.0273729.s002.docx]

**File S2. Live-cell imaging method for Figure 6H.**

Endothelial progenitor cells (EPC) and MCF10 A cells were grown in 96 well glass bottom plates (Greiner Bio- One). The cells were stained with Hoechst 33342, 2 µg/ml (Molecular Probes #H1399) in phenol-red free DMEM containing 5% FBS by incubating at 37°C for 10 minutes. Unbound dye was washed off by gently washing twice with PBS followed by staining the cells with mitochondrial transmembrane potential specific dye TMRM. Then the cells were treated with zerumbone 50 μM in media containing 5 nm of TMRM for imaging MMP loss. The same XY position was repeatedly imaged using Pathway Bio-imager 435 (Becton Dickinson, USA) at defined time periods using a macro created using the AttoVision 1.6 software. The images were acquired with 20 x 0.7 NA (Olympus) objective with 2x2 montage. The excitation/emission filters used for Hoechst is 350±50 nm and 460±60 nm for TMRM.
